# Efficacy and safety of therapeutic strategies for human brucellosis: A systematic review and network meta-analysis

**DOI:** 10.1371/journal.pntd.0012010

**Published:** 2024-03-11

**Authors:** Sarah Nascimento Silva, Gláucia Cota, Diego Mendes Xavier, Glaciele Maria de Souza, Marina Rocha Fonseca Souza, Moisés Willian Aparecido Gonçalves, Felipe Francisco Tuon, Endi Lanza Galvão

**Affiliations:** 1 Pesquisa Clínica e Políticas Públicas em Doenças Infecto-Parasitárias, Fundação Oswaldo Cruz, Belo Horizonte, Minas Gerais, Brazil; 2 Programa de Pós-Graduação em Reabilitação e Desempenho Funcional, Departamento de Fisioterapia, Universidade Federal dos Vales do Jequitinhonha e Mucuri, Diamantina Minas Gerais, Brazil; 3 Programa de Pós-Graduação em Odontologia Universidade Federal dos Vales do Jequitinhonha e Mucuri, Diamantina Minas Gerais, Brazil; 4 Programa de Pós-Graduação em Odontologia, Universidade Federal de Minas Gerais, Belo Horizonte, Minas Gerais, Brazil; 5 Programa de Pós-Graduação em Estomatopatologia da Universidade Estadual de Campinas, Campinas, São Paulo, Brazil; 6 Pontifícia Universidade Católica do Paraná, Curitiba, Paraná, Brazil; Mahidol Univ, Fac Trop Med, THAILAND

## Abstract

**Background:**

Human brucellosis is a neglected, re-emerging, and endemic zoonosis in many countries. The debilitating and disabling potential of the disease is a warning about its morbidity, generating socioeconomic impact. This review aims to update the current evidence on the efficacy and safety of therapeutic options for human brucellosis using the network meta-analysis (NMA).

**Methodology:**

A systematic search was conducted in four different databases by independent reviewers to assess overall therapy failure, adverse events, and time to defervescence associated with different therapies. Randomized clinical trials (RCTs) evaluating any therapeutic drug intervention were selected, excluding non-original studies or studies related to localized forms of the disease or with less than 10 participants. Data were analyzed by frequentist statistics through NMA by random effects model. The risk of bias and certainty of evidence was assessed, this review was registered at PROSPERO.

**Results:**

Thirty-one (31) RCTs involving 4167 patients were included. Three networks of evidence were identified to evaluate the outcomes of interest. Triple therapy with doxycycline + streptomycin + hydroxychloroquine for 42 days (RR: 0.08; CI 95% 0.01–0.76) had a lower failure risk than the doxycycline + streptomycin regimen. Doxycycline + rifampicin had a higher risk of failure than doxycycline + streptomycin (RR: 1.96; CI 95% 1.27–3.01). No significant difference was observed between the regimens when analyzing the incidence of adverse events and time to defervescence. In general, most studies had a high risk of bias, and the results had a very low certainty of evidence.

**Conclusions:**

This review confirmed the superiority of drugs already indicated for treating human brucellosis, such as the combination of doxycycline and aminoglycosides. The association of hydroxychloroquine to the dual regimen was identified as a potential strategy to prevent overall therapy failure, which is subject to confirmation in future studies.

## Author summary

Human brucellosis it is a disease with transmission associated with the handling of animals and ingestion of contaminated products. The disease usually presents with a subacute to chronic febrile condition characterized by nonspecific symptoms such weight loss, and sudoresis. Therapeutic regimens combining two or more drugs have been shown to be superior to monotherapy. The debilitating and disabling potential of the disease is a warning about its morbidity, whose symptoms can persist for weeks or months, generating socioeconomic impact. So far, the accumulated evidence is based on clinical trials that compared the interventions of interest against different comparators. The authors this reviewed all published studies and performed an analysis was capable of comparing the effect of therapeutic regimens to identify the best combinations to treat human brucellosis. The combination of three medicines (doxycycline + streptomycin + hydroxychloroquine) showed the best results compared to the option previously identified as more effective (doxycycline + streptomycin), so this research points to new perspectives for the treatment of brucellosis. This result point that more studies dedicated to studying this combination must be developed to confirm this trend and enable the adoption of new practices in the treatment of this disease.

## Introduction

Human brucellosis is the most prevalent bacterial disease in the world [1]. It is endemic mainly to countries in the Middle East, Asia, Africa, South and Central America, the Mediterranean Basin, and the Caribbean [1,2], and is considered a neglected and re-emerging disease [3].

The main *Brucella* species pathogenic to humans are *Brucella abortus and Brucella melitensis*, whose preferred hosts are, respectively, cattle and small ruminants (such as goats and sheep). Two other species are also associated with the human disease: *Brucella suis*, which has pigs as its preferred host, and *Brucella canis*, preferably related to the disease in dogs [4]. Transmission of the disease to humans occurs by contact of contaminated material with the conjunctiva or injured skin, ingestion of contaminated products, inhalation of bacteria, or accidental inoculation during animal vaccination [5,6]. Sporadic cases and outbreaks occur among consumers of unpasteurized dairy products, especially cheeses [7].

This acute or insidious disease is characterized by fever with a variable pattern, malaise, and night sweats, which can be associated with a peculiar moldy odor. Additional symptoms include weight loss, arthralgia, headache, low back pain, fatigue, anorexia, myalgia, cough, and emotional changes with depressive pattern [5,7]. In addition, human brucellosis can present with localized secondary manifestations, such as osteoarticular [8,9], genitourinary [10], cardiopulmonary [11], and neurological involvement, described in up to 10% of cases [11]. The debilitating and disabling potential represents an alarm about the disease’s morbidity, whose symptoms can persist for weeks or months, generating socioeconomic impact for individuals and communities [3].

Several classes of antimicrobials have been mentioned in the literature as valuable therapies in the treatment of brucellosis, including tetracyclines (doxycycline, minocycline), aminoglycosides (amikacin, gentamicin, and streptomycin), quinolones (ciprofloxacin and levofloxacin), as well as rifampicin, sulfamethoxazole/trimethoprim, and ceftriaxone [12]. Current accumulated evidence suggests the superiority of combined regimens over monotherapy [13]. To date, three systematic reviews have been conducted, combining therapeutic trials to treat the acute manifestations of brucellosis [12,14,15]. These reviews analyzed peer group analysis between interventions that included only dual regimens, one of which was restricted to comparing rifampicin versus streptomycin [14]. In recent years, new trials evaluating the efficacy and safety of treatments for human brucellosis [16,17] have been published, including interventions based on triple therapeutic regimens [18–20].

Given this context, the present study aimed to review the scientific literature systematically, analyzing the simultaneous comparison of multiple treatments to obtain an updated overview of the best therapeutic options used to treat the primary manifestation of human brucellosis.

## Methods

### Protocol and registry

This systematic review was conducted following the Cochrane Handbook [21] and the Preferred Reporting Items for Systematic Reviews and Meta-analyses (PRISMA) [22]. The study protocol was previously registered in PROSPERO (CRD42023411952).

### Eligibility criteria

The guiding question of this study was: "What is the efficacy and safety of therapeutic drug strategies for the treatment of human brucellosis?" The PICOS (population, intervention, comparator, outcome, study design) strategy was applied to select the studies: (P) patients with a confirmed diagnosis of human brucellosis; (I) any therapeutic drug intervention; (C) other therapeutic drug interventions, placebo, control; (O) overall therapy failure (combined outcome considering therapeutic failure/response to treatment + relapse + lost follow-ups); incidence of adverse events, and time to defervescence; (S) randomized clinical trials (RCTs). Secondary outcomes such as treatment adherence, quality of life, cost-effectiveness, and patient preference were also collected when available.

### Inclusion criteria

RCTs evaluating drug interventions for human brucellosis in patients diagnosed with the non-localized form of the disease were selected. The diagnosis was reached once at least one laboratory test (culture, agglutination, or molecular) and clinical signs confirmed the diagnosis of brucellosis.

### Exclusion criteria

The exclusion criteria were: (1) congress abstract whose results were reported in a scientific article (overlapping results); (2) studies reporting only localized form of human brucellosis; (3) studies that included less than 10 participants in each treatment arm; (4) publications in a language other than Portuguese, English or Spanish.

### Search strategy

The following electronic databases were searched: MEDLINE (PubMed), Embase, Cochrane Central Registry of Controlled Trials (CENTRAL), and Virtual Health Library (VHL). Articles published until January 6, 2023, were included. Studies published in English, Spanish, and Portuguese were included, and there were no restrictions on the date of publication. Additional studies were searched in the reference lists of the included articles. MeSH and EMTREE terms, keywords, and other free terms related to "human brucellosis," "treatment," and "clinical trial" were used with Boolean operators (OR, AND) to combine the search terms (S1 Table).

Searches were also conducted on two major platforms: ClinicalTrials.gov, provided by the U.S. National Library of Medicine, and https://opengrey.eu/, a Grey Literature Information system in Europe. In addition, searches were performed on the Google Scholar database (https://scholar.google.com/) with the main keywords defined in the search strategy.

### Selection process of the studies

Initially, the records obtained in the databases were exported to Mendeley Reference Management [23] to detect and eliminate duplicates and then transferred to the Rayyan application [24] for study selection. The records were evaluated independently and paired by two pairs of reviewers (GMS, DMX; MRFS, MWAG), both in terms of screening (reading titles and abstracts) and the reading phase of the full texts. Conflicts were discussed until a consensus was reached and, when necessary, were resolved by two other reviewers (ELG, SNS).

### Data extraction

Two researchers (ELG, SNS) performed data extraction by seeking information related to the main methodological characteristics, participants, and interventions for the general characterization of the included studies. This information was filled in spreadsheets in Microsoft Word, pre-specified, and validated by extracting pilot data from six articles.

### Outcome measures

The outcome of interest of this analysis was the incidence of intervention failure, commonly reported by failure occurrence and therapy relapse. Relapse cases were defined as those with the reappearance of signs and symptoms of the disease or positive laboratory results after completing therapy and passing through an asymptomatic period, a situation identified during patient follow-up. Therapeutic failure or non-response was defined as the persistence of signs and/or symptoms after treatment. Since therapy’s goal is improving disease manifestations from the beginning of treatment, the two outcomes were considered groupable, generating a overall therapy failure outcome. In this study, we considered cases of overall therapy failure, those without treatment response, with recurrence, and cases of lost follow-ups and treatment interruption. Eventual deaths were counted as lost follow-ups considering the initial number of participants recruited (intent to treat analysis).

### Risk of bias

The risk of bias for each outcome was assessed using the Revised Tool for Assessing Risk of Bias in Randomized Trials (RoB 2.0) [25] by two researchers (ELG, SNS) in an independent and paired manner. Thus, five domains were evaluated: randomization process, deviations from intended interventions, missing data, outcome measures, and selection of reported outcomes. After an independent assessment of the domains, an overall conclusion about the risk of bias was reached for each study.

A study was considered to have a low risk of bias when there was a low risk for all domains. A study was judged as having some concerns when at least one domain had some concerns, and no judgment of high risk of bias was identified. Finally, a study was judged as high risk of bias when at least one domain was considered to be at high risk.

### Meta-analysis

Initially, meta-analyses for binary comparisons were performed for comparisons evaluated in two or more studies, using relative risk (RR) as an effect measure for categorical data and mean difference (MD) as an effect measure for continuous data. Confidence intervals of 95% were used to present the results in all cases. Then, after similarity, heterogeneity, and consistency assumptions were confirmed, network meta-analyses (NMAs) were performed to allow the comparison of multiple treatments via a common comparator. In this case, the comparator chosen was doxycycline + streptomycin therapy for all outcomes. Frequentist statistics was used for all analyses with the random effects model. The analyses were conducted in R (https://cran.r-project.org) [26] with activation of the meta and netmeta packages [27].

The similarity was evaluated qualitatively, considering the methodological comparability between studies. The presence of heterogeneity was explored by consistency analysis between studies: the little overlap of confidence intervals for the results of individual studies indicated statistical heterogeneity. The I^2^ statistic was also used to assess heterogeneity between studies. Finally, the inconsistency was evaluated using the *netsplit* function.

Classification probabilities were estimated using the P-value, analog of the surface under the cumulative ranking curve (SUCRA) method used in Bayesian statistics, resulting in a probability ranking of each treatment being the best in relation to the others. Finally, a league table for each outcome was generated to obtain a comparison between all interventions.

To evaluate the combined outcome of combined failure, whenever possible, data were collected considering the intent to treat, and the losses observed during follow-up were considered therapeutic failures to compose this outcome. When the details of the number of follow-up losses were not provided by the treatment group, the analyses were performed considering the number of patients treated in each group (and not the randomized patients), and the study was penalized in the assessment of the risk of bias in the domain related to missing outcome data. The data were analyzed to evaluate adverse events, considering the population effectively treated with a specific intervention. To standardize the analyses, all times until defervescence were converted into hours.

### Certainty of evidence

The certainty of evidence was assessed for each domain using the Confidence in Network Meta-Analysis Software CINeMA [28,29]. Each estimate of the effect of the main therapies evidenced by the networks was evaluated according to the following criteria:

Within study bias—the risk of overall bias for each study was assigned according to the criteria of the RoB 2.0 tool. Next, each paired therapy comparison was evaluated based on the judgment of mean bias and the contribution of direct estimation from individual studies to the contribution matrix.

Indirectness—this domain considered whether the studies included in this review answered the research question targeted in terms of population, treatment, and outcome characteristics. We used a combined outcome as the primary outcome, although not all primary studies were addressed it in this way. Thus, studies reporting only one of the outcomes (relapse or therapeutic failure) were classified as moderate indirectness, and those that reported data comprising the complete outcome were classified as low indirectness. Each paired therapy comparison was ranked in the contribution matrix based on the judgment of mean bias, as follows.

Inaccuracy—we defined clinically important effects as a risk ratio lower than 0.8 and higher than its reciprocal 1.25.

Publication bias—evaluated by searching the gray literature and the international database for registration of clinical trials (Clinicaltrials.gov). In addition, we checked the sources of research funding to ensure no conflict of interest from the pharmaceutical industry contributing to publication bias.

Incoherence—we downgraded comparisons, resulting in statistical significance at the local node splitting test.

Heterogeneity—assessed by the agreement of the conclusions based on confidence and prediction intervals concerning the null and clinically important effects as 0.8.

Finally, we assigned an overall qualitative judgment to each comparison based on four levels of certainty of evidence: high, moderate, low, and very low. We downgraded the overall certainty of evidence starting from a high of one or two levels for the domains rated as "some concerns" or "major concerns," respectively.

## Results

### Systematic search

**Fig 1:**
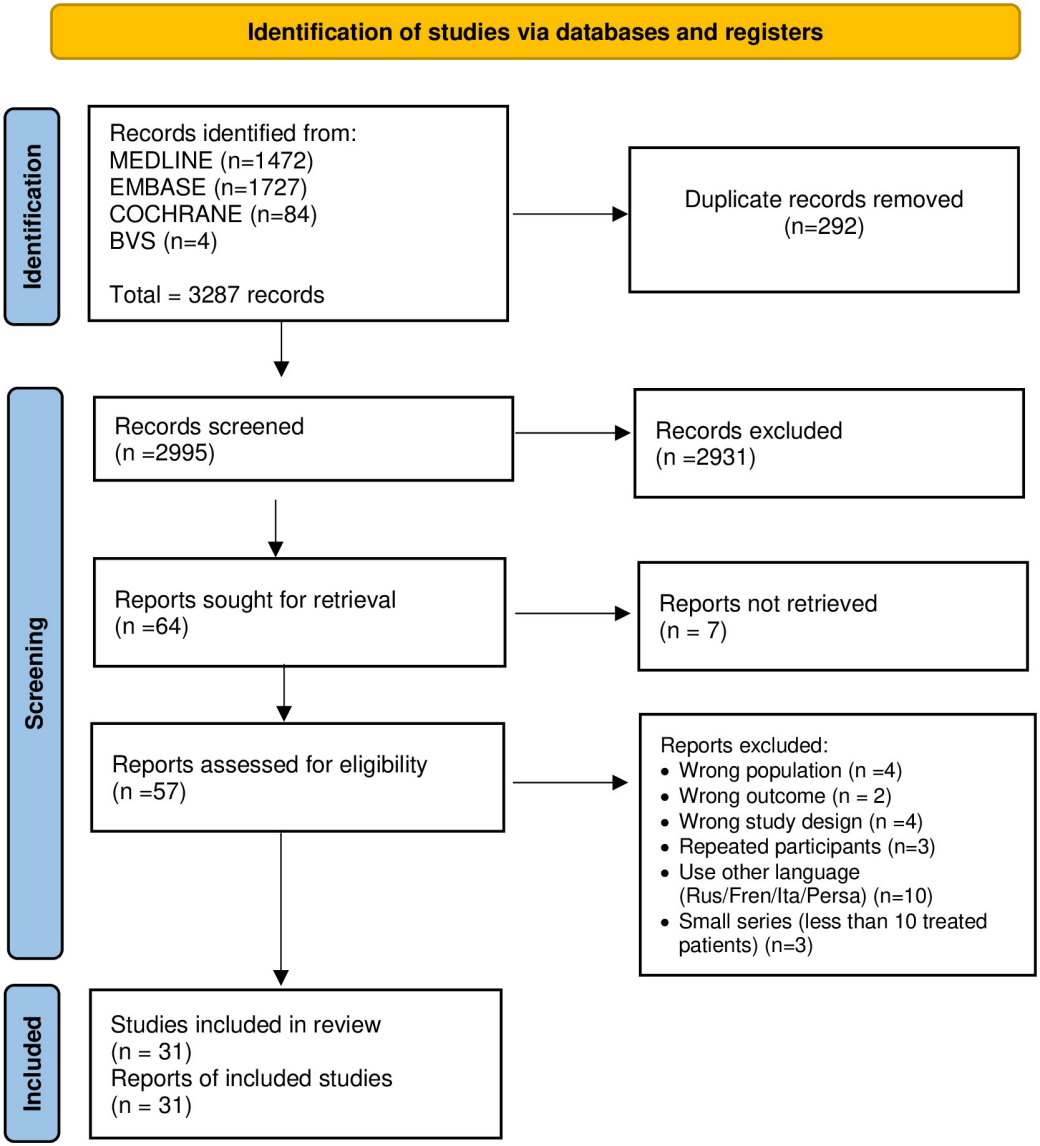
Flow chart of study selection.

Three thousand two hundred eighty-seven (3287) articles were identified in the databases. No additional studies were identified by searching the grey literature. After screening and selection based on eligibility criteria, 31 manuscripts [16–20,30–55] patients with human brucellosis were included. Fig 1 details the selection process in the PRISMA flowchart, and S2 Table summarizes the articles excluded in the full reading phase and the respective reasons for exclusion.

### Characteristics of the included studies

Tables 1 and 2 compile the main methodological characteristics of the included studies in terms of the studied population. S3 Table summarizes the definitions for the outcomes of interest adopted in the studies.

**Table 1:** Main methodological characteristics of the treatment of brucellosis studies (n = 31).

Year, Author	Country (cases)	Study arms (number of patients)	Follow-up (months)	Diagnostic criteria	Inclusion criteria	Exclusion criteria	Follow-up lost /treated patients
Feiz, 1973 [30]	Iran (95)	Group 1: DX Group 2: OXT+STP Group 3: OXT	3	Clinical signs and symptoms, a screening card teste, seroagglutination test and blood culture.	Patients with acute brucellosis.	NR	Both: 20/95
1982, Buzon [31]	NR (84)	Group 1: TE+RF Group 2:TMP/SMZ	6	Isolation of Brucella and/or seroconversion with compatible clinicals setting	Patients with brucellosis.	NR	NR
1985, Ariza [32]	NR (58)	Group 1: TE+STP Group 2: SMX-TMP	Group 1: 24 Group 2: 36	Cultures of blood in Castañeda medium were incubated for at least 6 weeks; Wright’s agglutination, Rose bengal, and Coombs antiglobulin tests.	Consecutive (Jan 1978 and Apr 1980) patients with brucellosis and cultures of blood positive for *B*. *melitensis* undergoing rigorous and prolonged clinical and bacteriologic follow-up.	NR	Both: 2/58
1987, Rodriguez Zapata [33]	Spain (72)	Group 1: DX+RF (34) Group 2: DX+STP (36)	12	Presence of a clinical picture compatible with acute brucellosis, Rose Bengal test positive and/or positive blood culture for Brucella.	Patients with acute brucellosis.	Age < 13y > 70y, pregnant women, severe concomitant diseases, patients requiring treatments with corticosteroids, barbiturates or other antibiotics, contraindication for any of the antibiotics used.	Group 1: 2/34 Group 2: 0/36
1989, Acocella [34]	Multicentric–France, Greece, Spain (146)	Group 1(A): DX+RF (63) Group 2 (B): DX+STP (53) Group 3 (C): TE+STP (27)	12	Clinical picture compatible with acute brucellosis and standard Tube agglutination Test above 125 IU, or complement fixation positive at a dilution of 1/8 or more, or positive blood culture for Brucella.	Combination of clinical symptoms and antibody levels, or from positive blood culture.	Age < 10y > 70y, pregnant women, severe concomitant diseases, allergic to any of the four antibiotics to be used.	Both: 3/146
1989, Colmenero [35]	Spain (111)	Group 1: DX+STP (59) Group 2: DX+RF (52)	6	(1) isolation of Brucella from blood or from any other body fluid and/or (2) clinical picture compatible with the disease together with (i) Wright’s séro agglutination at titres equal to or higher than 1/160; or (2) indirect immunofluorescence with titres equal to or higher than 1/100 for the IgS or IgG conjugates and equal to or higher than 1/50 for IgM or IgA conjugates, or (3) seroconversion of four or more times the initial titres in two separate serum samples taken with in a minimum interval of 3 weeks between them.	Patients diagnosed as suffering from brucellosis in our unit during 1985–1986.	Patients with neuromeningeal complications or those treated within the preceding 96 h with either tetracycline, streptomycin, rifampin, or co-trimoxazole.	Group 1: 0/59 Group 2: 0/52
1991, Solera [36]	Spain (84)	Group 1: DX+ RF (42) Group 2: DX+STP (42)	12	The diagnosis was made in 48 patients by isolating blood cultures of *Brucella sp*. and in the remaining 36 due to a clinical picture compatible with brucellosis plus a Wright’s serum agglutination titer greater than or equal to 1/160. The rose Bengal test was used as an auxiliary method (inclusion of patients with clinical signs that gave a positive result pending blood cultures or serum agglutination).	NR	Age < 7y; pregnant women; the patients who had taken some effective antimicrobial treatment in the 7 days before, severe concomitant disease, endocarditis, neurobrucellosis, patients with contraindications for taking any of the tested antibiotics. Patients admitted with a diagnosis of spondylitis.	Group 1: 8/42 Group 2: 8/42
1992, Ariza [37]	Spain (111)	Group 1: DX+RF (53) Group 2: DX+STP (51)	12	Positive culture to *Brucella melitenses* or clinical findings and titer of antibodies to Brucella STAT (>1/160).	Positive culture or clinical findings and standard tube agglutination	Endocarditis or neurobrucellosis.	Group 1: 9/53 Group 2: 7/58
1993, Akova [38]	Turkey (61)	Group 1: DX+RF (30) Group 2: OFX+RF (31)	12	Serological detection of antibodies to *Brucella sp*. (STAT>1/160) and compatible clinical findings and isolation of a *Brucella sp*.	Patients diagnosed as suffering from brucellosis according to diagnostic criteria.	Pregnant women, endocarditis, neurobrucellosis, individuals who received antimicrobial therapy prior to the study, patients allergic to any of the drugs employed in the regimens.	0
1993, Montejo [39]	Spain (375)	Group 1: DX+RF (4 weeks) Group 2: SMX-TMP (6 months) Group 3: DX (6 weeks) Group 4: STP (2 or 3 weeks) + DX 6 weeks Group 5: DX+RF (6 weeks) Group 6: DX+STP (6 weeks)	12	Isolation of germs from clinical specimens, and/or titers of the standard tube agglutination test (STAT) of 1/160.	≥ 14 years of age, a clinical picture consistent with a diagnosis of brucellosis, isolation of germs from clinical specimens, and/or titers of the standard tube agglutination test (STAT) of 1/160.	Pregnant women, antecedents of brucellosis in the previous year, the presence of serious associated illness, a reported allergy to one or more of the antimicrobial agents used in this study, diagnosis of endocarditis, spondylitis, or affection of the CNS by Brucella.	Both: 45/375
1994, Colmenero [40]	Spain (20)	Group 1: DX+STP (9) Group 2: DX+RF (10)	6	Isolation of the organism or clinical and serological Symptoms (seroagglutination ≥1/160 and anti-Brucella Coombs test≥1/320).	Normal hepatic and renal functions; received any drugs or took any alcohol during the treatment period.	NR	0/20
1995, Solera [41]	Spain (194)	Group 1: DX+RF (100) Group 2 (DS): DX+STP (94)	12	Isolation of Brucella species or titer of antibodies to Brucella *sp*. (STAT > 1/160) with compatible clinical findings	Patients diagnosed as suffering from brucellosis according to diagnostic criteria.	Pregnant women, nursing; known or suspected hypersensitivity to or another contraindication for tetracyclines, rifampicins, or aminoglycosides; severe concomitant disease; and effective antimicrobial therapy within 7 days before entry into the study.	Group 1: 13/100 Group 2: 14/94
1996, Kalo [42]	Albania (24)	Group 1: DX+CIP (12) Group 2: DX+RF (12)	6	Positive serology and/or isolation of a *Brucella sp*. from blood in the presence of compatible epidemiological and clinical findings. The serological criteria were the following: Wright seroagglutination assay titers equal to or higher than 1/160 and/or indirect immunofluorescence assay titers higher than 1/100.	NR	Age < 15y, pregnant women, individuals who received antimicrobial therapy prior to the study, patients allergic to the drugs employed, patients with brucellosis and severe complications of this disease, such as central nervous system involvement, endocarditis or spondilitis.	Group 1: 0/12 Group 2: 0/12
1999, Agalar [43]	Turkey (40)	Group 1: DX+RF (20) Group 2: CIP+RF (20)	12	*Brucella sp*. infection based on clinical and laboratory findings (blood cultures)	Patients suspected to have Brucella infection based on clinical and laboratory findings.	Age < 15 years, history of seizures, recent antibiotic use, allergy to the study antibiotics, and pregnancy	Group 1: 0/20 Group 2: 0/20
2002, Saltoglu [44]	Turkey (57)	Group 1: DX+RF (30) Group 2: OFX+RF (27)	6	Isolation of *Brucella sp*. in blood, body fluids, and compatible clinical picture supported by the detection of specific antibodies at significant titers and demonstration of an at least 4-fold rise, or both in antibody titer in serum specimens had been taken after 2 weeks.	Patients with brucellosis had been followed at the Clinical Microbiology and Infectious Diseases Department, Çukurova University, Balcalı Hospital, Adana, Turkey, between January 1997 and February 2001.	NR	NR
2004, Solera [45]	Spain (146)	Group 1: DX+GT (84) Group 2: DX+GT (83)	12	Isolation of the organism or serological detection of antibodies to *Brucella sp*. (STAT ≥1:160; or a ≥4-fold increase in the Brucella antibody titer to >1:80, revealed by standard tube agglutination, between serum specimens obtained ≥2 weeks apart and studied in the same laboratory)	Age ≥18 years with brucellosis diagnosed and at least 2 of the following compatible clinical findings must have been present: fever, arthralgias, weight loss, hepatosplenomegaly, or signs of focal disease.	Known or suspected hypersensitivity (or another contraindication) to tetracyclines or aminoglycosides, severe concomitant disease, body weight of ≤50 kg, or receipt of effective antimicrobial therapy for brucellosis≤ 30 days before entering the study.	Group 1: 7/80* Group 2: 8/81*
2004, Karabay [46]	Turkey (34)	Group 1: DX+RF (18) Group 2: OFX+RF (16)	3	Presence of signs and symptoms compatible with brucellosis including a positive agglutination (titre≥1/160) and/or a positive culture	Patients diagnosed as suffering from brucellosis according to diagnostic criteria.	Age < 15y, history of seizure, pregnant women.	Group 1: 4/18 Group 2:1/16
2004, Roushan [47]	Iran (280)	Group 1: DX+SMX-TMP (140) Group 2: SMX-TMP +RF (140)	12	≥1/320 standard tube agglutination titer (STAT) of antibodies to *Brucella sp*. with a 2 mercaptoethanol (2 ME) ≥1/160, in association with compatible clinical findings. Confirmatory tests were also performed using Elisa with significant titers of IgM and IgG specific Brucella antibodies.	Consecutive patients with brucellosis attended from April 1999 to January 2002 in the Department of Infectious Diseases, Yahyanejad Teaching Hospital, Babol Medical University.	Age < 10y, pregnant women, spondylitis, endocarditis, meningoencephalitis, previous history of brucellosis, and antimicrobial therapy for more than 7 days before enrollment.	Group 1: 10/140 Group 2: 23/140
2005, Ersoy [48]	Turkey (128)	Group 1: OFX+RF (41) Group 2: DX+RF (45) Group 3: DX+STP (32)	6	(1) Isolation of *Brucella sp*. from blood or other fluids, or (2) the finding of ≥1/160 titre or four-fold rise over 2–3 weeks in titre of antibodies to Brucella by a standard-tube agglutination test in association with characteristic clinical findings, and history of consuming unpasteurized or raw milk.	Ambulatory and hospitalized patients between May 1997 and December 2002, newly diagnosed as uncomplicated brucellosis.	Pregnant women, nursing, known or suspected hypersensitivity or any contraindication to rifampicines, tetracyclines or aminoglycosides, severe concomitant disease and effective antimicrobial therapy within 10 days before starting the study.	Both: 10/128
2006, Roushan [49]	Iran (200)	Group 1 (DS): DX+STP (100) Group 2: DX+GT (100)	12	STAT ≥1:320 and 2ME ≥1:80 who had clinical findings compatible with this diagnosis.	Patients diagnosed as suffering from brucellosis according to diagnostic criteria.	Age <10y, spondylitis, neurobrucellosis, pregnant women, and receipt of 11 week of antibiotic treatment before enrollment.	Group 1: 6/100 Group 2: 3/100
2007, Alavi [50]	Iran (105)	Group 1: DX+RF (52) Group 2: DX+SMX-TMP (53)	6	Compatible clinical findings and finding significant titers (STAT ≥1/80 with 2-ME ≥1/40.	Patients diagnosed as suffering from brucellosis according to diagnostic criteria.	Age < 15y, pregnant women, spondylitis, endocarditis, meningoencephalitis, previous history of brucellosis, antimicrobial therapy for more than seven days before enrollment.	Group 1: 1/52 Group 2: 2/53
2007, Ranjbar [51]	Iran (228)	Group 1: DX+RF (114) Group 2: DX+RF + AM (114)	6	(1) Brucellosis clinical features including fever, sweats, arthralgia, hepatomegaly, splenomegaly, and/or signs of focal disease with a ≥1/160 standard tube agglutination titer of antibodies to Brucella; or (2) a tissue sample or blood culture positive for Brucella bacteria; or (3) a four-fold increase in Wright titer in a two-week interval with compatible clinical findings.	Consecutive patients with brucellosis who attended the Hamedan Sina Hospital between 1999 and 2001, whether seen as outpatients or as inpatients	Age < 8y, pregnant women, endocarditis and neurobrucellosis.	Group 1: 4/114 Group 2: 4/114
2009, Keramat [52]	Iran (178)	Group 1: DX+RF (61) Group 2: CIP+RF (62) Group 3: CIP+DX (55)	6	Symptoms compatible with acute brucellosis with titer of antibodies to Brucella (STAT ≥1/160 and 2-ME ≥1/80, and/or a positive blood culture.	Patients diagnosed as suffering from brucellosis according to diagnostic criteria.	Age <17y, pregnant women, meningitis, neurobrucellosis, endocarditis, renal or hepatic failure	Group 1: 0/61 Group 2: 0/62 Group 3: 0/55
2009, Sarmadian [53]	Iran (80)	Group 1: DX+RF (40) Group 2: DX+RF+ CIP (40)	6	NR	Patients over 13 years old and attended one of the two infectious disease clinics in Arak, Iran between 2006–2008.	NR	NR
2010, Roushan [54]	Iran (164)	Group 1: DX+STP (82) Group 2: DX+GT+DX (82)	12	Standard tube agglutination (STA) titre ≥1:320 and 2-mercaptoethanol (2ME) titre ≥1:160, together with compatible clinical findings (fever, sweating, arthralgias, peripheral arthritis, sacroiliitis and epididymo-orchitis).	Outpatient and inpatient cases aged > 10 years.	Spondylitis, endocarditis, neurobrucellosis, pregnant woman, and those who had received antibiotics for > 2 days.	Group 1: 5/82 Group 2: 2/82
2012, Hashemi [17]	Iran (219)	Group 1: OFX+RF (73) Group 2: DX+RF+LEV (73) Group 3: DX+STP (73)	6	Clinical presentation and significant titers of specific antibodies (STAT ≥1/160, Coombs test ≥1/160, 2-ME ≥1–80, or Brucella IgG-ELISA >12) and/or a positive blood culture.	Patients diagnosed as suffering from brucellosis according to diagnostic criteria.	Age <17 years, endocarditis, neurobrucellosis, spondylitis, renal or hepatic failure, or treatment for brucellosis in the last 6 months.	Group 1: 9/73 Group 2: 11/73 Group 3: 8/73
2014, Sofian [18]	Iran (158)	Group 1: DX+RF+STP 6 weeks (79) Group 2: DX+RF+STP 8 weeks (79)	24	Compatible signs and symptoms, standard tube agglutination test (STA) ≥ 1:160, and 2-mercaptoethanol (2ME) agglutination ≥ 1:80.	Patients over 9 y old with a diagnosis of uncomplicated brucellosis.	Pregnant women and patients with other underlying disorders.	Group 1: 7/79 Group 2: 7/79
2016, Hasanain [19]	Egypt (107)	Group 1: DX+RF(60) Group 2: DX+RF+LEV(60)	6	Contact with animals or fresh animal products and suggestive clinical manifestations of less than one-year duration, and positive antibody titer (>1:160).	Patients with acute/subacute brucellosis who had not received any antimicrobial therapy since the start of illness.	Pregnant and pediatric patients	Group 1: 7/60 Group 2: 6/60
2018, Majzoobi [16]	Iran (177)	Group 1: DX+STP+HC (89) Group 2: DX+STP (88)	6	Compatible clinical feature with either a positive Brucella serology [Wright ≥1/160, 2-mercaptoethanol (2ME) ≥ 1/80] or positive blood or bone marrow cultures for Brucella.	Patients aged at least 18 years and represented the criteria of acute brucellosis, including a compatible clinical feature with either a positive Brucella serology [Wright ≥1/160, 2-mercaptoethanol (2ME) ≥ 1/80] or positive blood or bone marrow cultures for Brucella	Patients with glucose- 6-phosphate dehydrogenase deficiency, pregnant women, significant preexisting diseases (porphyria, psoriasis, macular degeneration), or other severe hematological, gastrointestinal, or neurological diseases.	Group 1: 0/89 Group 2: 0/88
2020, Karami [55]	Iran (339)	Group 1: DX+RF+GT (175) Group 2: DX+RF+GT (164)	6	Clinical and serological symptoms of the selected patients (Wright>1.80; 2ME>1.40).	Age >12 years; confirmed diagnosis; absence of complications; admission in Valiars Hospital.	(1) Allergic reactions to medications; (2) History of renal diseases; (3) Hearing and balance disorders.	NR
2022, Majzoobi [20]	Iran (92)	Group 1: DX (200 mg for 4 weeks) (46) Group 2: DX (200 mg for 6 weeks) (46)	6	Clinical presentations consistent with brucellosis with positive serology including standard tube agglutination (Wright) test ≥ 1/160 and 2-mercaptoetanol (2ME) ≥ 1/80.	Adult inpatients with brucellosis.	Age < 18y, pregnant women, neurobrucellosis, endocarditis, spondylitis, serious complication of brucellosis, patients who did not want to continue clinical and laboratory follow-up.	Group 1: 0/46 Group 2: 0/46

DX: Doxycycline; RF: Rifampicin; STP: Streptomycin; OFX: Ofloxacine; GT: Gentamicin; LEV: Levofloxacin; HC: Hydroxychloroquine; CIP: Ciprofloxacin; CFX: Ceftriaxone; SMX-TMP: Sulfametoxazol-trimetoprim; AM: amikacin; TE: Tetracylcline, OXT: Oxytetracycline; STAT: Standard Tube Agglutination Test; 2 ME: 2-mercaptoethanol, NR: not reported, y: Years.

* Patients without diagnostic confirmation were not counted as losses

**Table 2:** Characteristic of the population enrolled in the treatment of brucellosis studies and therapy schedules (n = 31).

Year, Author	Intervention (mg/frequency/duration) (n)	Comparison (mg/frequency/duration) (n)	Treatment length	Age (mean±SD, years)/ (range)	Gender male/female	Clinical manifestations (n or %)	Clinical form (n)	Brucella species Characterization (n)	Mean ± SD (range) duration of symptomatic (days)
1973, Feiz [30]	Group 1: DX/400mg/daily/14 days and 200mg/daily/7 days (31)	Group 2: OXT 25-30mg/kg + STP 20mg/kg/ daily/14 days (28) Group 3: OXT 25-30mg/kg/daily/21 days (16)	Group 1: 21 days Group 2: 14 days Group 3: 14 days	NR	NR	NR	Acute (95)	NR	NR
1982, Buzon [31]	Group 1: TE/500mg + RF 1.200mg/daily during the first week and 600mg/daily during following 3 weeks/over 4 weeks (46 courses)	Group 2: TMP/SMZ (480/2400mg (10days), 320/1600mg(20days) and 160/400mg until the end of treatment for 6 months (46 courses)	Group 1: 4 weeks Group 2: 6 months	NR	NR	NR	Active brucellosis (84)	NR	NR
1985, Ariza [32]	TE /0.5g/21 days + STP /1g/daily/14 days (27)	SMZ/TMP /240 to 1,200mg/twice daily/45 days (28)	Group 1: 21 days Group 2: 45 days	Group 1: 32.6 (13–72) Group 2: 32.3 (7–69)	Group 1: 20/71 Group 2: 25/3	NR	NR	*B*. *melitensis* (NR)	NR
1987, Rodriguez Zapata [33]	DX /200mg/daily/45 days + RF /900mg/daily/45 days (34) (ITT36)	DX/200mg/daily/21 days + STP IM/1g/daily/21 days (36) (ITT36)	Group 1: 45 days Group 2: 21 days	NR	Group 1: 28/6 Group 2: 28/8	Fever (70), Joint pain (59), Sweating (59), Asthenia (51), Splenomegaly (39), Headache (34), Hepatomegaly (32), Anorexia (26), Myalgia (20), Lymph node enlargement (19), Sacroiliitis (9), Radiculitis (7), Constipation (6), Peripherical arthritis (5), Orchitis (4), Spondilitis (2), Insomnia (2), Meningitis (1)	Acute	NR	NR
1989, Acocella [34]	Group 1: RF 900 mg/daily + DX/200mg/daily/45 days (63)	Group 2: DX /200 mg/daily/45 days + STP IM/ 1g/daily/21 days (53) Group 3: TE /2g/daily/ (0–5 g 4x daily)/21 days STP IM/ 1g/daily/14 days (27)	Group 1: 45 days Group 2: 45 days Group 3:21 days	Group 1: 39 Group 2:40 Group 3:45	Group 1: 36/27 Group 2: 41/12 Group 3:17/20	Splenomegaly (31%), lymphnode enlargement (24%), localized disease (14%)	Acute brucellosis (146)	*B*. *melitenses* (68)	NR
1989, Colmenero [35]	DX/100 mg /12h/30 days (59) + STP IM/1g/21 days (52)	DX /100mg/12h + RF /15mg/Kg/45 days	Group 1: 21 days Group 2: 45 days	Group 1: 33.46±5.26 Group 2: 32.74±2.15	Group 1: 45/14 Group 2: 32/20	Hepatomegaly (43), Splenomegaly (27)	Focal forms (40)	*B*. *melitensis (57)*	NR
1991, Solera [36]	DX/200mg/daily/45days + RF 900 mg/daily/21days (42)	DX/200mg/daily/45days + STP IM/ 1g/daily/14 days (42)	Group 1: 45 days Group 2: 45 days	Both: 32.1±16.6 Group 1: 34±17 Group 2: 29±15	Both: 65/17 Group 1: 32/10 Group 2: 33/9	Artrite (24), Fever (72), Sweating (72), Astenia (64), Perda de peso (35), Artralgias (48), Mialgias (35), Dor lombar (51), Cefaleia (44), Sintomas digestivos (28), adenopatia (12), hepatomegalia (19), esplenomegalia (28)	Focal (29)	NR	Group 1: 28±38 Group 2: 17±15
1992, Ariza [37]	Group 1: DX/100mg/twice/45days + RF /15mg/kg/45 days (44) (ITT 53)	Group 2: DX/100mg/45 days STP IM/1g/14 days (94)	45 days	39,1±16,5 Group 1 36,8±15,9 Group 2 41,9±116,6	Group 1: 31/13 Group 2: 37/14	NR	Focal disease (16)	*B*. *Melitensis (81)*	Group 1: 37,0 ±31,1 Group 2: 49,4 ± 59,1
1993, Akova [38]	Group 1: DX/200mg/daily/6 weeks + RF /600mg/daily/6 weeks (30)	Group 2: OFX /400mg/daily/6 weeks + RF /600mg/daily/6 weeks (31)	Group 1:45 days Group 2: 30 days	Group 1: 34,4 ± 14,3 Group 2: 37,8 ± 15,1	Group 1:16/14 Group 2: 14/17	NR	NR	*B*. *melitensis* (49)	Group 1: 31,4 ± 21,7 Group 2: 37,1 ±24,5
1993, Montejo [39]	Group 1: RF /1,200mg/daily/7 days and then 600 mg/d for 21 days + DX /200mg/daily/28 days (65)	Group 2: TMP /160mg + SMX /800mg/8h/10 days; 12h until 4 weeks; TMP/80mg+SMX/400mg until 6 months (64) Group 3: DX/200mg/day/6 weeks (71) Group 4: STP IM/1g/day/3weeks + DX /200mg/daily/6 weeks (44) Group 5: RF/900mg/daily + DX /200mg/daily/6 Weeks (46) Group 6: STP IM/1g/daily/2 weeks + DX /200mg/daily/6 Weeks (40)	Group 1: 28 days Group 2: 6 months Group 3: 6 weeks Grous 4: 6 weeks Group 5: 6 weeks Group 6: 6 weeks	Both: 46 (14–82)	Both: 242/88	Fever (313), Perspiration (297), Chills (269), Arthralgia (211), Constitutional symptom (181), Hepatomegaly (93), Splenomegaly (56), Adenopathy (26), Sacroiliitis (27), Orchitis (14), Skin lesions (7)	NR	*B*. *melitensis (66)* *B*. *abortus (3)*	NR
1994, Colmenero [40]	Group 1: DX /100mg/ 12 h/6 weeks + STP IM/1g/daily/3 weeks (10)	Group 2: DX plus RF: 600 mg/daily (weighed between 50 and 60 kg) 900 mg/daily (weighed between 61 and 80 kg) 1,200 mg/daily (weighed more than 80 kg) (10)	6 weeks	Both: 33.3 ± 15.6 (17–69) Group 1: 35,3±18,8 Group 2: 32,1±12,1	Group 1: 7/3 Group 2: 6/4	NR	Focal disease (8)	*B*. *melitensis* (12)	60.2 ± 55.7 Group 1: 67,5±73,2 Group 2: 53,7±35,8
1995, Solera [41]	Group 1: DX/5mg/kg of body weight per day if the body weight was 40 kg or less/twice/45 days + RF/900mg/daily/45 days (100)	Group 2: DX + STP IM/1g/daily/14 days (94)	Group 1:45days Group 2: 45 days	Group 1: 33 (7–77) Group 2: 34 (12–70)	Group 1: 80/20 Group 2: 79/15	NR	Focal disease (78)	*B*. *melitensis* (NR)	Group 1: 27 ± 34 Group 2: 25 ± 29
1996, Kalo [42]	Group 1: CIP/1,000mg/day/6 weeks + DX /200mg/day/6 weeks (12)	Group 2: RF/900/mg/day/6 weeks + DX/200/ mg/day/6 weeks (12)	Group 1: 6 weeks Group 2: 6 weeks	Both: 31.76±13.5 (18–56)	Both: 14/10	NR	Acute	*B*. *melitensis (11)*	Both: 21.42±14.52
1999, Agalar [43]	Group 1: DX/100mg/twice/ + RF /600mg/daily/45 days (20)	Group 2: CIP/500mg/twice + RF/600mg/daily/30 days (20)	Group 1:45 days Group 2: 30 days	Group 1: 37.1±15.8 Group 2: 37.8±13.9	Group 1:13/7 Group 2:8/12	Fever 39 (97.5%), headache 16 (40%), sweating 35 (87.5%), arthralgia 35 (87.5%), myalgia 31 (77.5%), arthritis 6 (15%), sacroiliitis 3 (7.5%), epididymoorchitis 1 (2.5%)	NR	NR	Group 1: 36.45±28* Group 2: 24.35±16.1*
2002, Saltoglu [44]	Group 1: RF 600mg/daily/45 days + DX 100mg/BID (30)	Group 2: OFX/200mg/twice/45 days RF/ 600mg/daily (27)	Group 1: 2 weeks Group 2: 2 weeks	Both: 36.8 ±11.3 (15–65)	Group 1: 8/22 Group 2: 6/21	Fever (49), Arthralgia (49), Hepatomegaly (18), Splenomegaly (38), Arthritis (12), Sacroiliitis (15)	NR	NR	NR
2004, Solera [45]	Group 1: DX/100mg/twice/30 days + Placebo GT/240mg/daily/7 days (73) (84 ITT)	Group 2: DX /100mg/twice/45 days + GT/240mg/daily/7 days (73) (83 ITT)	Group 1: 30days Group 2: 45days	Group 1: 38.3± 13.7 Group 2: 39.5± 15.4	Group 1: 62/9 Group2: 60/13	NR	Focal disease (26) Group 1: 14 Group 2: 12	NR	Group 1: 30.2 ±28.3 Group 2: 33.5±33.4
2004, Karabay [46]	Group 1: DX 100mg/twice/45 days + RF 600mg/ daily/45 days (14) (18 ITT)	Group 2: OFX 400mg/daily/30 days RF+ 600mg/daily/30 days (15) (18 ITT)	Group 1: 45 days Group 2: 30 days	Group 1: 29 (24–61) Group 2: 35 (18–60)	Group 1: 11/3 Group 2: 13/2	Group 1: fever (13), artharalgia (10) Headache (10) Group 2: fever (13), artharalgia (10) Headache (10)	NR	NR	NR
2004, Roushan [47]	Group 1: CTX 8mg/kg/day of the trimethoprim component divided into 3 doses + DX 100mg/ twice daily (140)	Group 2: CTX 8mg/kg/day of the trimethoprim component divided into 3 doses + RF 15mg/kg/daily (140)	Group 1: 2 months Group 2: 2 months	Group 1: 35.56±16.2 (12–81) Group 2: 31.39±17.88 (10–79)	Group 1: 74/66 Group 2: 76/64	Arthralgia (185), Myalgia (94), Splenomegaly (19)	Acute (208), Subacute and chronic arthritis (72)	NR	NR
2005, Ersoy [48]	Group 1: OFX 400mg/daily/6 weeks + RF 600mg/daily/6 weeks (41)	Group 2: DX 200mg/daily/6 weeks + RF 600mg/daily/6 weeks (45) Group 3: DX 100mg/daily/6 weeks + STP IM 1g/3 weeks (32)	Group 1: 6 weeks Group 2: 6 weeks Group 3: 6 weeks	Group 1: (16–70) Group 2: 18–75 Group 3: 17–62	Group 1: 23/18 Group 2: 24/21 Group 3: 15/17	NR	NR	NR	Group 1: 23.7± 2.2 Group 2: 25.3±1.7 Group 3: 28.4±2.5
2006, Roushan [49]	Group 1: STP IM/1g/14 days + DX 100mg/twice/45 days (94) (100 ITT)	Group 2: GT 5 mg/kg/daily/7 days + DX 100mg/twice/45 days (97) (100 ITT)	45 days	Group 1: 36.2±14.14 Group 2: 33.74±16.6	Group 1: 52/42 Group 2: 57/40	Group 1: Fever 69 (73.4%); Sweating 80 (85.1%); Arthralgia 67 (71.3%) Group 2: Fever 75 (77.3%); Sweating 91 (93.8%); Arthralgia 71 (73.2%)	Acute (154), Focal disease (51)	NR	NR
2007, Alavi [50]	Group 1: DX /100mg/ twice RF /300mg/8 hour (51) (52 ITT)	Group 2: DX/100mg/ twice + CTX /2 adult tablets or 960mg/twice (51) (53 ITT)	8 weeks	Group 1: 31.26 ±11.23 Group 2: 29.89 ±9.81	NR	Group 1: Fever 29 (56.86); Sweating 40 (78.43); Arthralgia 40 (78.43); Low back pain 48 (94.11) Group 2: Fever 28 (54.90); Sweating 41 (80.39); Arthralgia 41 (80.39); Low back pain 49 (96.07)	NR	NR	NR
2007, Ranjbar [51]	Group 1: DX 100mg/twice daily + RF 10mg/kg/daily/8 weeks (114)	Group 2: DX 100mg/twice daily + RF 10mg/kg/daily/8 weeks + AM IM/7.5mg/kg/twice daily/7 days (114)	Group 1: 21 days Group 2: 45 days	Group 1: 37±18.4 Group 2: 35.7±17	Both: 107/113 Group 1: 53/57 Group 2: 54/56	Fever (203)	NR	*B*. *melitensis* (16) Negative blood culture (69)	NR
2009, Keramat [52]	Group 1: DX 200 mg/daily + RF 15 mg/kg/daily (600–900 mg) orally (61)	Group 2: CIP 15 mg/kg/daily (500–750 mg twice a day) + RF 15 mg/kg/daily (62) Group 3: CIP 15 mg/kg/daily (500–750 mg twice a day) + DX 200 mg/daily (55)	8 weeks**	Group 1: 39.67 Group 2: 43.66±NR Group 3: 38.41	Group 1: 43/18 Group 2: 34/28 Group 3: 38.41	Malaise (157), arthralgia (142), generalized pain (140), sweating (140) and headache (128).	Acute (178)	NR	NR
2009, Sarmadian [53]	Group 1: DX/ 100 mg/BID/8 weeks + RF/10 mg/kg/daily/8 weeks (40)	Group 2: DX/ 100mg/BID/8 weeks + CIP/500mg/BID/8 weeks (40)	Group 1: 8 weeks Group 2: 8 weeks	NR	NR	NR	NR	NR	NR
2010, Roushan [54]	Group 1: GT/5 mg/kg/daily/5 days + DX /100mg/twice daily/8 weeks (82)	Group 2: STP IM/1g/2 weeks + DX/1g/45 days (82)	Group 1: 8 weeks Group 2: 45 days	Group 1: 35.9±14.8 Group 2: 36.5±14.5	Both: Group 1: 56/82 Group 2: 52/82	Fever, Sweating, Arthralgia, Peripheral arthritis, Sacroiliitis, Epididymo-orchitis, Spondylitis	Acute (164)	NR	NR
2012, Hashemi [17]	Group 1: OFX 800 mg daily + RF 15 mg/kg daily for 6 weeks (64) (ITT = 73)	Group 2: DX 200 mg/ daily + RF 15mg/kg/ daily/6 weeks (62) (ITT = 73) Group 3: DX 200mg daily/6 weeks + STP 1,000mg/ Daily/3 weeks (65) (ITT = 73)	6 weeks	Group 1: 40.5± 14.2 Group 2: 38.6 ± 17.3 Group 3: 39.9± 15.4	Group: 1:36/28 Group: 2:34/28 Group: 3:26/29	Arthralgia (74.3%), fatigue (73.8%), low back pain (68.1%), body ache (57.6%), sweating (49.2%), headache (46.6%), and anorexia (45.0%)	NR	*B*. *melitensis* (12)	Group 1: 60.1 ± 103 Group 2: 68.6 ± 150.9 Group 3: 46.6 ± 34.3
2014. Sofian [18]	Group 1: DX 100 mg/twice daily/6 weeks + RF 600 mg/daily/ 6 weeks + STP IM 750–1,000 mg/ daily/7 days (79)	Group 2: DX 100 mg/twice daily/8 weeks + RF 600 mg/daily/ 8 weeks + STP IM 750–1,000 mg/ daily/7 days (79)	Group 1: 6 weeks Group 2: 8 weeks	Both: 35.9±15 Group 1: 37.7±14.6 Group 2: 34.2±15.18	Both: 93/53 Group 1: 43/29 Group 2: 48/24	Fever (68.6%), arthralgia (62.6%), anorexia (53.5%), night sweating (52.9%), malaise (49.3%), myalgia (42%), headache (25%), cough (10.4%), diarrhea (2.8%).	Uncomplicated brucellosis	NR	9.5 days (3–40)
2016, Hasanain [19]	Group 1: DX 200mg/daily + RF 900 mg/daily/6 weeks (53) (ITT = 60)	Group 2: DX 200mg/daily + RF 900mg/daily + LEV 500mg/daily/6 weeks (54) (ITT = 60)	6 weeks	Group 1: 32.7 ± 15 Group 2: 36.2 ± 17.4	Group 1: 32/21 Group 2: 30/24	Fever 103 (96.3%), Fatigue 89 (83.2%), Myalgia 75 (7.1%), Chills 71 (66.4%), Sweats 66 (61.7%), Arthralgia 52 (48.6%), Relative bradycardia 34 (31.8%), Splenomegaly 23 (21.5%), Hepatomegaly 18 (16.8%), Lymphadenopathy 15 (14%)	Acute (NR), Subacute brucellosis (NR)	NR	NR
2018, Majzoobi [16]	Group 1: HC 6.5 mg/kg + DX 100 mg/twice daily/6 weeks + STP 1000 mg/day (<60 years) or 750 mg/day (aged at least 60 years)/3 weeks (89)	Group 2: DX 100 mg/twice daily/6 weeks + STP 1000 mg/day (<60 years) or 750 mg/day (aged at least 60 years)/3 weeks (88)	6 weeks	Group 1: 38.5±17.2 Group 2: 42.5±6.4	Group 1: 59/30 Group 2: 58/30	Generalized pain, Arthralgia, Headache, Fever, Arthritis, Spondylitis, Orchitis	Acute (177)	NR	NR
2020, Karami [55]	Group 1: DX tablets (100 mg twice daily for 8 weeks) + RF (600 mg/d for 8 weeks, and gentamicin (5 mg/kg/d for 7 d). (175)	Group 2: DX tablets (100 mg twice daily for 12 weeks) + RF (600 mg/d for 12 weeks + GT (5 mg/kg/d for 7 d)(164)	Group 1: First group: 8 weeks Group 2: Second group:12 weeks	45.95 ± 18.65	Both: 193/146	Fever (81.1%), sweating (65.8%), myalgia (69%), arthralgia (56.9%), headaches (14.5%), lethargy (46%), loss of appetite (44.5%), coughs (8%), diarrhea (4.1%), and dysuria (13/6%).	NR	NR	Mean 30 (21–56) days
2022, Majzoobi [20]	DX 200 mg/daily/4 weeks + HC 400 mg/daily/4 weeks + STP 1 g/daily/3 weeks (46)	DX 200 mg/daily/6 weeks + HC 400 mg/daily/6 weeks + STP 1 g/daily/3 weeks (46)	Group 1: 28 days Group 2: 42 days	Group 1: 37.4±5 Group 2: 40.11±5	Group 1: 35/11 Group 2: 29/17	Myalgia (74), Headache (37), Arthralgia (54), Fever (62)	NR	NR	NR

DX: Doxycycline; RF: Rifampicin; STP: Streptomycin; OFX: Ofloxacine; GT: Gentamicin; LEV: Levofloxacin; HC: Hydroxychloroquine; CIP: Ciprofloxacin; CFX: Ceftriaxone; SMX-TMP: Sulfametoxazol-trimetoprim; AM: amikacin; TE: Tetracylcline, OXT: Oxytetracycline; STAT: Standard Tube Agglutination Test; 2 ME: 2-mercaptoethanol, NR: not reported, y: Years.

* Duration of fever (in days) before admission to hospital

** Those with spondylitis or unresponsive to an eight-week therapy received additional four-week treatment

### Meta-analysis

**Fig 2:**
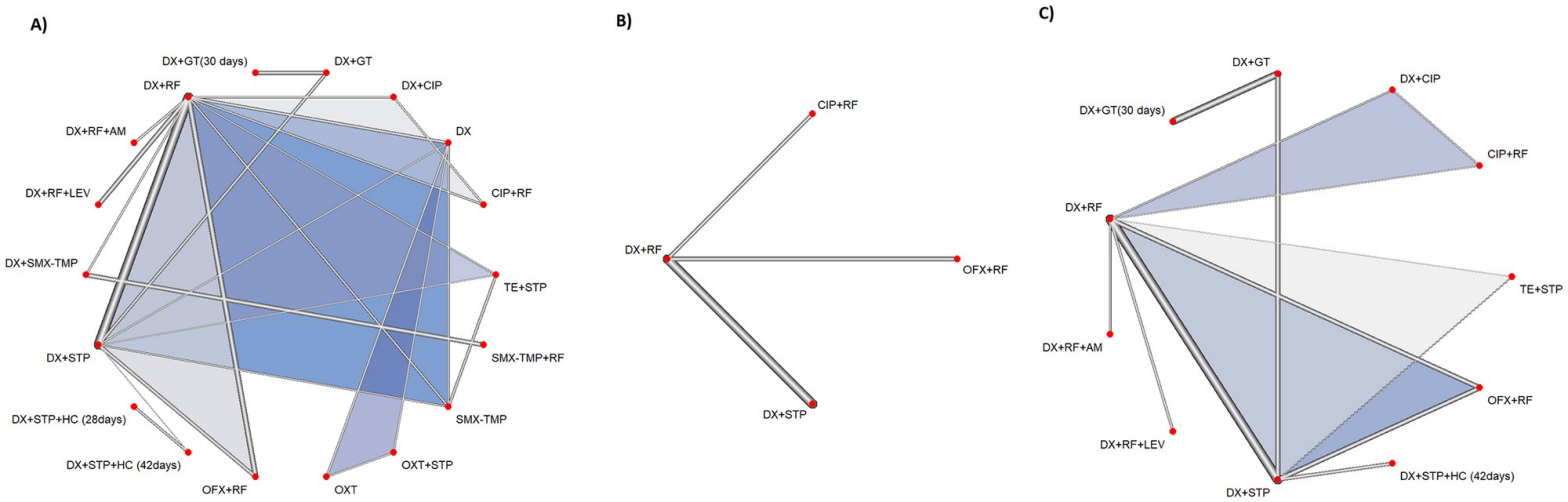
Network plot of the outcomes (A) Overall therapy failure; (B) Time to defervescence; (C) Adverse events.

Three outcomes were considered of interest for comparing therapeutic interventions in human brucellosis: incidence of overall therapy failure, time to defervescence, and incidence of adverse events with different treatments. The outcome most described in the literature as a proxy for efficacy was failure with a given intervention (inverted outcome). Fig 2 illustrates the networks of evidence with the therapies evaluated for each outcome. All networks met the principles of homogeneity, similarity, and consistency. Given the impossibility of identifying a single network of evidence for each outcome, some studies could not be part of the analyses [16,47,54] because they tested combinations of therapies that were not compared with those included in the networks. While Sofian et al. (2014) [18] compared the combination of doxycycline + rifampicin (six weeks) + streptomycin versus doxycycline + rifampicin (eight weeks) + streptomycin, Karami et al. (2020) [55] compared doxycycline + rifampicin + gentamicin used at eight and 12 weeks of treatment. Therefore, these studies could not comprise any of the analyses. The study by Roushan (2004) [47] as the only one that compared sulfamethoxazole/trimethoprim (SMX/TMP) + rifampicin therapies and reported results for adverse events; thus, although it had to be excluded from the network of this outcome, it could be part of the network that evaluated overall therapy failure.

The intervention used as a comparator in the analyses was doxycycline + streptomycin. This is the most frequently used intervention of the doxycycline-aminoglycoside group, representing the current treatment indication for the disease.

### Overall therapy failure

Twenty-eight (28) studies involving 3217 participants and 20 intervention types reported the outcome of relapse after some improvement with treatment as the measure of failure. On the other hand, 18 studies involving 2256 participants and 12 intervention types showed non-response to treatment. Thus, these studies contributed to the NMA for the combined outcome of treatment failure.

**Fig 3:**
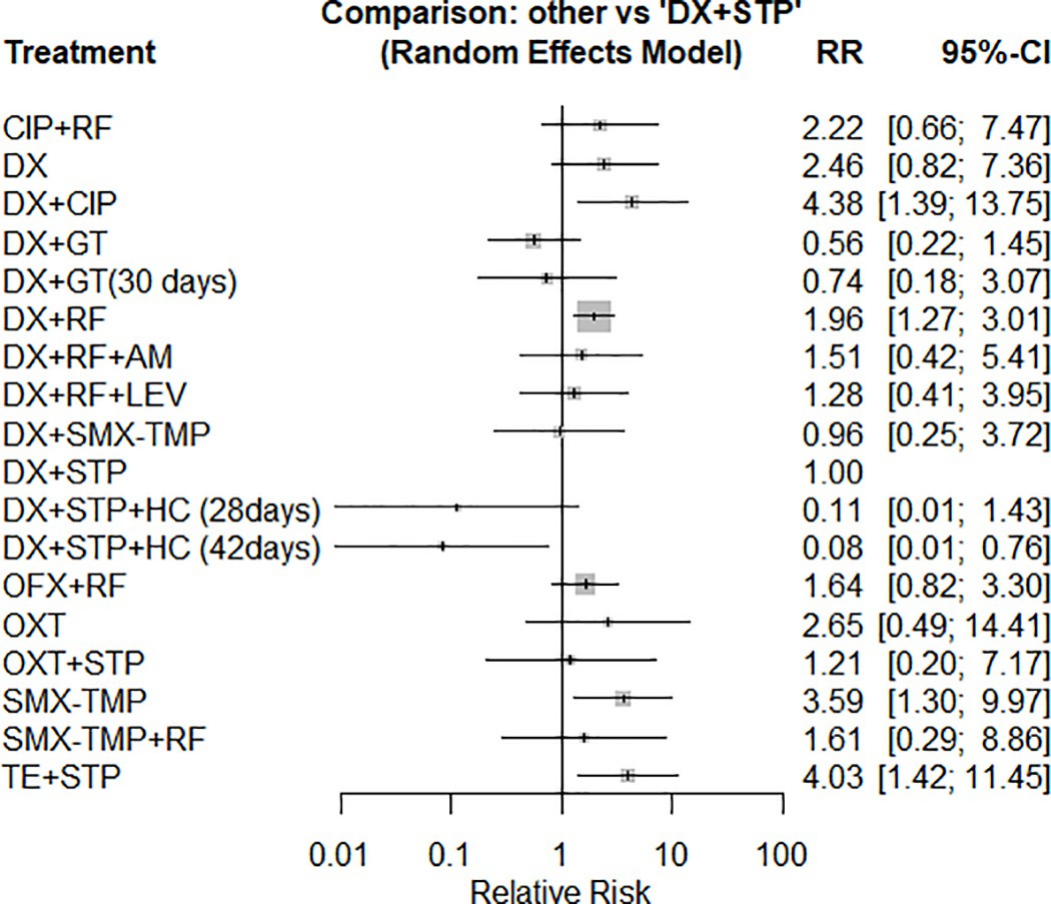
Forest plot of network meta-analysis of the overall therapy failure outcome (all schemes against doxycycline + streptomycin regimen).

Only triple therapy with doxycycline + streptomycin + hydroxychloroquine used for 42 days (RR: 0.08; CI95% 0.01 to 0.76; very low certainty–S4 Table) had a protective effect against overall therapy failure compared to the doxycycline + streptomycin regimen. There was no statistically significant difference between the doxycycline + streptomycin regimen and doxycycline + gentamicin (RR: 0.56; CI 95% 0.22 to 1.45; very low certainty–S4 Table). The doxycycline + rifampicin regimen had a higher risk of overall therapy failure than doxycycline + streptomycin (RR: 1.96; CI 95% 1.27 to 3.01; very low certainty–S4 Table). Fig 3 shows all comparisons conducted against the latter therapy.

When all treatments were compared with the others in a league table (Table 3), doxycycline + gentamicin had a lower risk of failure than doxycycline + rifampicin (RR: 0.30; CI 95% 0.14 to 0.62; very low certainty–S4 Table).

**Table 3:** League table of network meta-analysis: Relative risk (RR) for overall therapy outcome.

**CIP+RF**																	
1.14(0.36;3.63)	**DX**																
0.44 (0.19;1.00)	0.38 (0.13;1.13)	**DX+CIP**															
3.87 (1.18;12.70)	3.39 (1.27;9.04)	8.82 (2.90;26.81)	**DX+GT**														
2.95 (0.80;10.95)	2.59 (0.84;7.97)	6.73 (1.94;23.30)	0.76 (0.44;1.33)	**DX+GT(30days)**													
1.15 (0.45;2.94)	1.01 (0.51;1.97)	2.62 (1.13;6.07)	0.30 (0.14;0.62)	0.39 (0.16;0.97)	.**DX+RF**												
1.49 (0.44;5.07)	1.31 (0.47;3.67)	3.40 (1.08;10.74)	0.39 (0.13;1.12)	0.51 (0.15;1.69)	1.30 (0.59;2.84)	**DX+RF+AM**											
1.76 (0.61;5.08)	1.54 (0.67;3.55)	4.01 (1.51;10.63)	0.45 (0.19;1.10)	0.60 (0.21;1.68)	1.53 (0.93;2.51)	1.18 (0.47;2.97)	**DX+RF+LEV**										
2.34 (0.64;8.62)	2.05 (0.67;6.32)	5.34 (1.56;18.32)	0.61 (0.19;1.93)	0.79 (0.22;2.87)	2.04 (0.83;5.02)	1.57 (0.48;5.18)	1.33 (0.48;3.73)	**DX+SMX-TMP**									
2.23 (0.84;5.91)	1.95 (0.96;3.96)	5.08 (2.11;12.24)	0.58 (0.29;1.14)	0.76 (0.31;1.82)	1.94 (1.50;2.51)	1.49 (0.66;3.40)	1.27 (0.73;2.22)	0.95 (0.37;2.43)	**DX+STP**								
19.69 (1.81;213.67)	17.24 (1.75;169.89)	44.86 (4.29;469.19)	5.09 (0.52;49.77)	6.67 (0.64;69.66)	17.13 (1.91;153.31)	13.18 (1.29;135.04)	11.20 (1.18;105.95)	8.40 (0.79;89.90)	8.83 (1.00;77.81)	**DX+STP+HC(28days)**							
27.07 (2.88;254.60)	23.70 (2.79;201.14)	61.69 (6.82;557.71)	7.00 (0.83;58.89)	9.17 (1.01;82.80)	23.55 (3.08;180.22)	18.12 (2.05;160.31)	15.40 (1.90;125.06)	11.55 (1.25;107.02)	12.14 (1.61;91.36)	1.37 (0.61;3.10)	**DX+STP+HC(42days)**						
1.59 (0.58;4.41)	1.39 (0.64;3.03)	3.63 (1.43;9.18)	0.41 (0.18;0.92)	0.54 (0.20;1.44)	1.39 (0.93;2.05)	1.07 (0.44;2.56)	0.91 (0.48;1.71)	0.68 (0.25;1.82)	0.71 (0.46;1.10)	0.08 (0.01;0.74)	0.06 (0.01;0.46)	**OFX+RF**					
1.06 (0.24;4.62)	0.93 (0.37;2.31)	2.42 (0.59;9.91)	0.27 (0.07;1.05)	0.36 (0.08;1.53)	0.92 (0.30;2.87)	0.71 (0.18;2.81)	0.60 (0.18;2.08)	0.45 (0.11;1.93)	0.48 (0.15;1.51)	0.05 (0.00;0.63)	0.04 (0.00;0.40)	0.67 (0.20;2.21)	.**OXT**				
2.32 (0.48;11.14)	2.03 (0.70;5.87)	5.29 (1.17;23.98)	0.60 (0.14;2.54)	0.79 (0.17;3.69)	2.02 (0.57;7.09)	1.55 (0.35;6.82)	1.32 (0.34;5.10)	0.99 (0.21;4.65)	1.04 (0.29;3.72)	0.12 (0.01;1.47)	0.09 (0.01;0.93)	1.46 (0.39;5.43)	2.19 (0.68;7.00)	**OXT+STP**			
0.69 (0.21;2.22)	0.60 (0.25;1.44)	1.56 (0.52;4.68)	0.18 (0.07;0.48)	0.23 (0.07;0.73)	0.60 (0.30;1.21)	0.46 (0.16;1.32)	0.39 (0.17;0.92)	0.29 (0.09;0.92)	0.31 (0.15;0.64)	0.03 (0.00;0.35)	0.03 (0.00;0.22)	0.43 (0.19;0.96)	0.65 (0.18;2.29)	0.30 (0.07;1.17)	**SMX-TMP**		
1.39 (0.35;5.57)	1.22 (0.36;4.13)	3.17 (0.85;11.89)	0.36 (0.10;1.26)	0.47 (0.12;1.85)	1.21 (0.44;3.36)	0.93 (0.26;3.37)	0.79 (0.26;2.46)	0.59 (0.37;0.95)	0.62 (0.22;1.79)	0.07 (0.01;0.79)	0.05 (0.01;0.50)	0.87 (0.29;2.61)	1.31 (0.29;6.02)	0.60 (0.12;3.02)	2.03 (0.59;6.99)	**SMX-TMP+RF**	
0.70 (0.21;2.34)	0.62 (0.24;1.60)	1.61 (0.52;4.95)	0.18 (0.07;0.51)	0.24 (0.07;0.77)	0.61 (0.29;1.30)	0.47 (0.16;1.39)	0.40 (0.16;0.99)	0.30 (0.09;0.97)	0.32 (0.15;0.68)	0.04 (0.00;0.36)	0.03 (0.00;0.23)	0.44 (0.19;1.03)	0.66 (0.18;2.48)	0.30 (0.07;1.26)	1.03 (0.52;2.04)	0.51 (0.14;1.79)	**TE+STP**

DX: Doxycycline; RF: Rifampicin; STP: Streptomycin; OFX: Ofloxacine; GT: Gentamicin; LEV: Levfloxacin; HC: Hydroxychloroquine; CIP: Ciprofloxacin; CFX: Ceftriaxone; SMX-TMP: Sulfametoxazol-trimetoprim; AM: amikacin; TE: Tetracylcline, OXT: Oxytetracycline.

**Fig 4:**
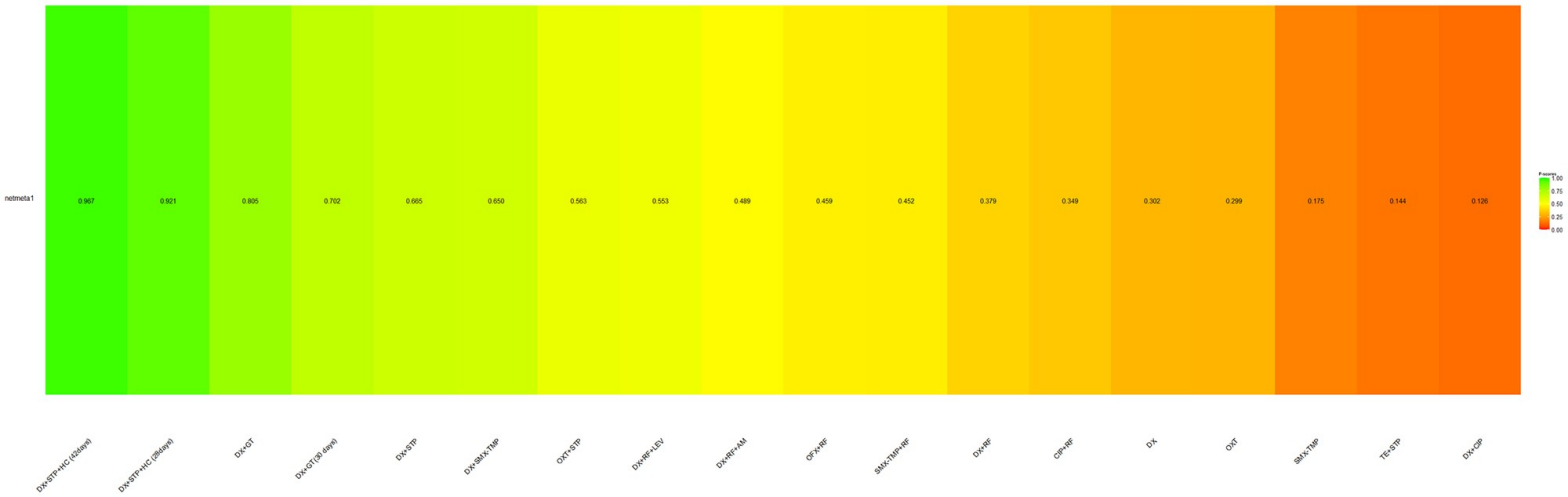
Frequentist analogue of SUCRA performed for overall therapy failure outcome.

Fig 4 illustrates the classification of interventions for treating human brucellosis and their likelihood of being the best or worst in relation to the risk of overall therapy failure. Therapies with doxycycline+ streptomycin + hydroxychloroquine used for 42 and 28 days were most likely to avoid failure considering the direct and indirect comparison together (P-score = 0.967 and 0.921, respectively). The third best therapy in the ranking was doxycycline + gentamicin (P-score = 0.805), while the worst therapies were SMX-TMP monotherapy (P-score = 0.144) and doxycycline + ciprofloxacin (P-score = 0.126).

No inconsistency was detected in the NMA for the overall therapy failure outcome (Q = 3.70; P = 0.977) or statistical heterogeneity (I^2^ = 0%; tau = 0).

### Time to defervescence

**Fig 5:**
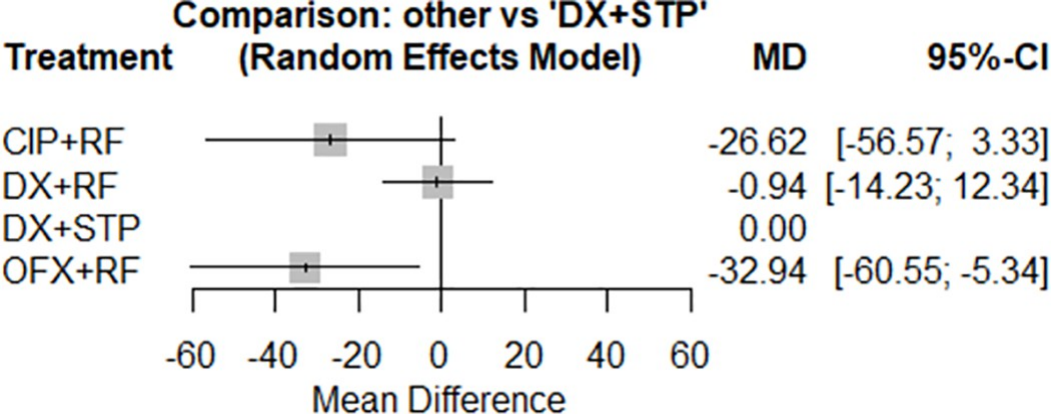
Forest plot of network meta-analysis of the time to defervescence outcome (all schemes against doxycycline + streptomycin regimen).

Only five studies, with 379 participants and four categories of interventions contributed to the NMA that evaluated the time to defervescence. There was no difference in this time between the therapies with doxycycline + streptomycin versus doxycycline + rifampicin and ciprofloxacin + rifampicin (MD: -26.62; CI95%, -56.57 to 3.33 hours), although treatment with ofloxacin + rifampicin (MD: -32.94; CI95%, -60.55 to -5.34 hours) significantly reduced the fever duration (Fig 5).

When comparing all treatments, doxycycline + rifampicin and doxycycline + streptomycin showed significantly longer time to defervescence compared to ofloxacin + rifampicin (MD: 32.00; CI95% 11.60 to 52.40 hours; MD: 33.51; CI95% 10.44 to 56.58 hours, respectively).

In ranking the best-performing probabilities, doxycycline + streptomycin was in the worst position (P-score = 0.165). The inconsistency could not be detected since there was no closed loop in the network of this outcome [56–58].

### Adverse events

Twelve studies (12) involving 1819 participants and 11 intervention types were evaluated in NMA for the outcome incidence of adverse events.

**Fig 6:**
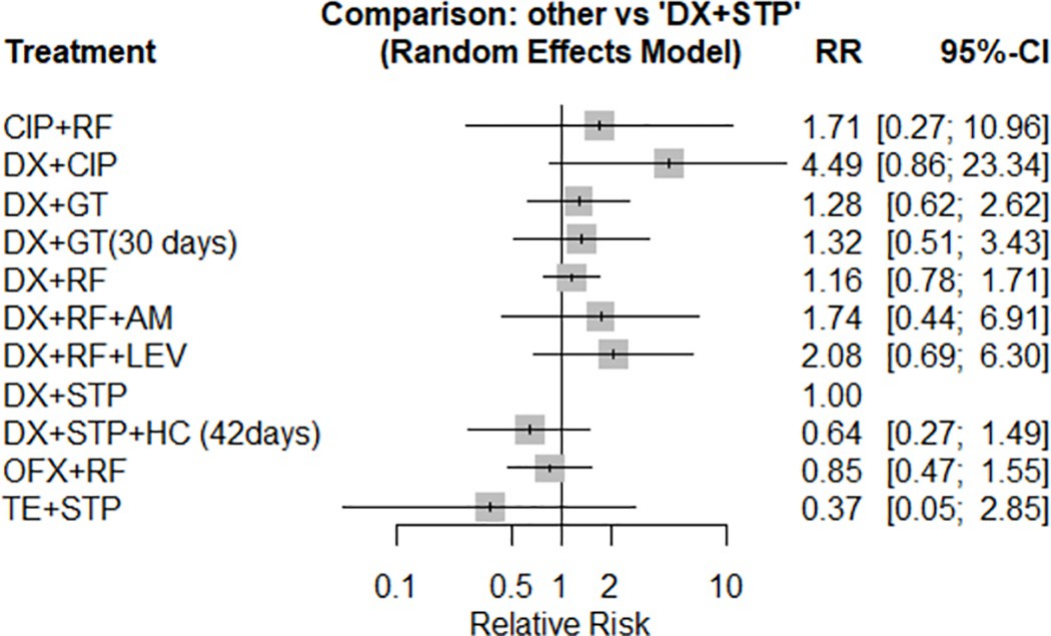
Forest plot of network meta-analysis of adverse events outcome (all schemes against doxycycline + streptomycin regimen).

There was no significant difference in the risk of adverse events between the therapies compared to the doxycycline + streptomycin (Fig 6). When all treatments were compared (Table 4), the doxycycline + gentamicin regimen showed no difference in the incidence of adverse events (very low certainty of evidence, S5 Table).

**Table 4:** League table of network meta-analysis: Relative risk (RR) for adverse events outcome.

**CIP+RF**										
0.38 (0.10;1.40)	**DX+CIP**									
1.41 (0.22;9.05)	3.71 (0.71;19.28)	**DX+GT**								
1.36 (0.20;9.13)	3.58 (0.66;19.55)	0.97 (0.64;1.45)	**DX+GT(30days)**							
1.48 (0.26;8.53)	3.88 (0.84;17.90)	1.05 (0.56;1.94)	1.08 (0.52;2.27)	**DX+RF**						
0.98 (0.12;8.42)	2.59 (0.36;18.49)	0.70 (0.18;2.78)	0.72 (0.17;3.06)	0.67 (0.19;2.30)	**DX+RF+AM**					
0.82 (0.11;5.94)	2.16 (0.36;12.84)	0.58 (0.19;1.76)	0.60 (0.19;1.96)	0.56 (0.22;1.39)	0.83 (0.18;3.89)	**DX+RF+LEV**				
1.80 (0.30;10.69)	4.74 (1.00;22.53)	1.28 (0.75;2.18)	1.32 (0.68;2.59)	1.22 (0.90;1.66)	1.83 (0.51;6.55)	2.20 (0.83;5.79)	**DX+STP**			
2.81 (0.42;19.07)	7.40 (1.34;40.89)	2.00 (0.83;4.82)	2.07 (0.78;5.45)	1.91 (0.89;4.09)	2.86 (0.67;12.25)	3.43 (1.04;11.34)	1.56 (0.78;3.14)	**DX+STP+HC (42days)**		
2.11 (0.34;13.01)	5.54 (1.11;27.57)	1.49 (0.72;3.11)	1.55 (0.67;3.58)	1.43 (0.88;2.32)	2.14(0.57;8.09)	2.57 (0.91;7.27)	1.17 (0.71;1.93)	0.75 (0.32;1.77)	**OFX+RF**	
4.83 (0.34;69.27)	12.70 (1.02;157.98)	3.43 (0.43;27.16)	3.55 (0.43;29.26)	3.27 (0.44;24.28)	4.91 (0.47;51.75)	5.89 (0.65;53.42)	2.68 (0.36;19.81)	1.72 (0.21;14.27)	2.29 (0.29;17.83)	**TE+STP**

DX: Doxycycline; RF: Rifampicin; STP: Streptomycin; OFX: Ofloxacine; GT: Gentamicin; LEV: Levofloxacin; HC: Hydroxychloroquine; CIP: Ciprofloxacin; CFX: Ceftriaxone; SMX-TMP: Sulfametoxazol-trimetoprim; AM: amikacin; TE: Tetracylcline, OXT: Oxytetracycline.

**Fig 7:**
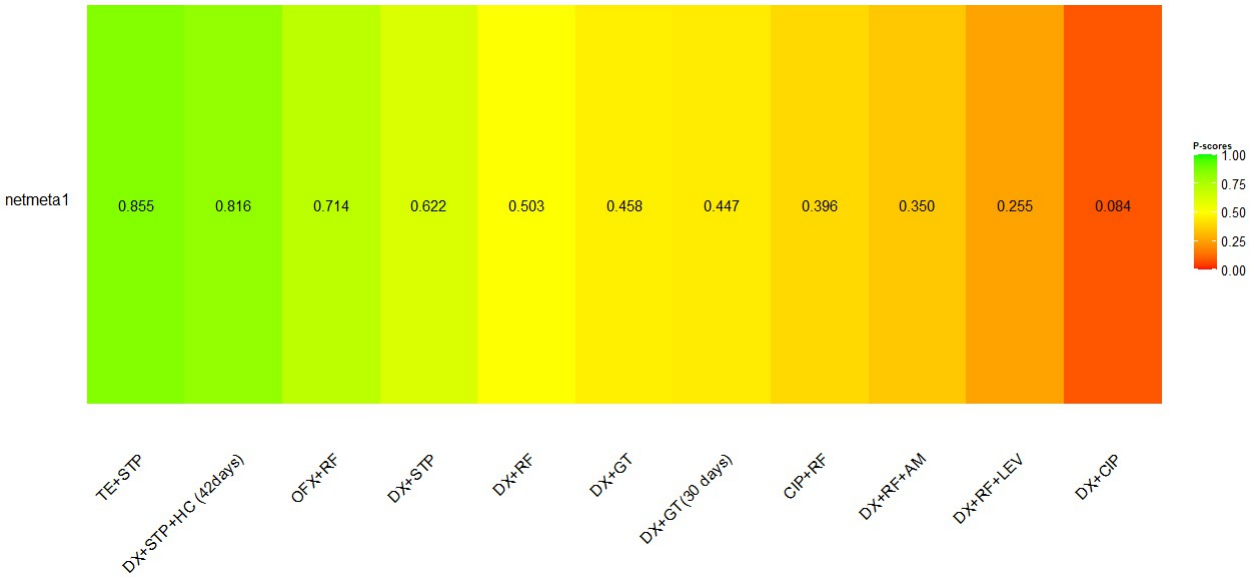
Frequentist analogue of SUCRA performed for adverse events outcome.

Fig 7 illustrates the classification of interventions for treating human brucellosis and their likelihood of causing adverse events. Treatment regimens with tetracyclin + streptomycin and doxycycline+ streptomycin + hydroxychloroquine used for 42 days were related to the lower probability of causing adverse events (P-score = 0.855 and 0.816, respectively). The therapies with the highest probability of adverse events were doxycycline + rifampicin + levofloxacin (P-score = 0.255) and doxycycline + ciprofloxacin (P-score = 0.084).

No inconsistency was detected in the NMA for the adverse outcome events (Q = 6.23; P = 0.182) or statistical heterogeneity (I^2^ = 0%; tau = 0).

### Assessment of risk of bias

In general, the information available in the studies for most domains was incomplete or missing. The definition of the outcome and the measurement methodology were the parameters related to a higher risk of bias in evaluating the combined treatment failure and occurrence of adverse events outcomes (most studies had a high risk of bias; S6 and S7 Tables). As for the outcome time to defervescence, the main weakness was the lack of blinding and detailing of the outcome measure (S8 Table).

## Discussion

This review updates the synthesis of evidence available for therapeutic interventions for treating human brucellosis with the differential of using a more comprehensive approach based on indirect comparisons made possible by network meta-analyses. The results are in line with those of previous studies, confirming the superiority of the doxycycline + streptomycin regimen for six weeks compared to doxycycline + rifampicin [12,15]. Therapeutic regimens based on binary combinations, including doxycycline, predominate in the literature. Three types of therapeutic approaches based on the combination of three drugs were identified, namely doxycycline + rifampicin + amikacin, doxycycline + rifampicin + levofloxacin, and doxycycline + streptomycin + hydroxychloroquine. This last association investigated in a study published in 2018 [16] stands out, as it showed a protective effect compared to other regimens for the outcome of overall therapy failure.

In this review, a more comprehensive outcome for evaluating the outcome of therapeutic intervention in brucellosis was defined, bringing together the absence of response and relapse, as adopted in the review conducted by Pozo et al. (2012) [15]. Using this combined outcome was a useful strategy to gather a greater number of studies and strategies to allow the approximation with the outcome of greater clinical relevance, that is, to estimate the unfavorable evolution after each treatment as an inverted proxy of the efficacy measure. Although the data are reported, the studies do not always use this combination of outcomes to measure the performance of the therapeutic regimen. While some articles present this measure together [47,49], others show the results based only on disease recurrence [45,46,55]. In addition, NMA modeling allowed the comparison of multiple treatment regimens through simultaneous analysis of results. The certainty the evidence was assessed for each comparison [56–58], which qualifies the results obtained, although it points to the methodological weaknesses found.

Six new studies carried out in the last 10 years were added to the set of available evidence, five of which evaluated the response to triple therapies [16,18–20,55]. A study conducted in 2014 compared two durations of triple therapy with doxycycline + rifampicin + streptomycin and found no difference in the occurrence of relapse between treatments withn six or eight weeks [18]. Karami et al. (2020) [55], evaluating the same intervention in 2020 in eight and 12 weeks, also did not identify the superiority of the longer treatment in relation of recurrence. In the findings of this review and those published previously [12,15], the superiority of aminoglycoside regimens is well established. However, the use of triple therapy remains a factor under evaluation, either by proving the superiority or even indicating the appropriate treatment time. The results of Sofian [18] and Karami^55^ still leave a gap to be investigated: the comparison of the doxycycline-rifampicin-streptomycin regimen for six and 12 weeks of treatment. These findings should include, in addition to the efficacy of the treatment, aspects such as adverse event profile, cost, and adherence to the therapeutic regimen, since they considerably influence long-term therapies, like those used in the treatment of brucellosis.

In 2016, Hasanain et al. [19] compared the classic doxycycline + rifampicin regimen with triple therapy with the addition of levofloxacin; the authors found that this last regimen reduced the incidence of relapse. The use of antimicrobials of the quinolone class in the treatment of brucellosis has been tested in many studies [38,43,44,46,48]. Although no significant difference was observed in the direct comparison between quinolone-rifampicin versus doxycycline-rifampicin regimens in the review conducted by Pozo et al., 2012 [15], our network analyses suggest a higher probability of overall therapy failure of ciprofloxacin therapy. The regimens with levofloxacin and ofloxacin are related to intermediate probabilities of overall therapy failure, while regimens including aminoglycosides exhibit superior efficacy than regimens using quinolones.

Mojzoobi et al. [16,20] evaluated the triple therapy regimen with doxycycline-streptomycin-hydroxychloroquine in two studies. This choice was based on previous *in vitro* and in *vivo* studies that related the proliferation and survival of *Brucella sp*. to an acidic environment interfering with the disease virulence [59]. In 2018, the six-week triple regimen was compared to doxycycline-streptomycin and associated with lower relapses [16]. In 2022, the authors compared success with the triple therapy doxycycline and hydroxychloroquine for four or six weeks associated with streptomycin for three weeks, and found no differences between groups [20]. Our results suggest the superiority of the triple regimen in terms of success probability, including six weeks with hydroxychloroquine over doxycycline + streptomycin. It is worth emphasizing that the certainty of this comparison’s evidence was very low, and therefore, it should be interpreted with caution. A recent review concluded that the use of triple therapy is superior to double regimens, but none of the studies analysed included hydroxychloroquine regimens [60]. Furthermore, the comparator used in most dual therapy studies of this review [60] (doxycycline + rifampicin) has a higher overall therapy failure rate than the comparator used in our analysis (doxycycline + streptomycin).

Although the study with triple therapy with hydroxychloroquine had a considerable number of participants (n = 177) and did not report serious adverse events, it should be noted that this is a single study evaluating such association [16]. The toxicity of hydroxychloroquine found in other clinical indications [61], should also be emphasized, reinforcing the need for further studies before its incorporation into clinical practice for treating human brucellosis. The rationale for the association of hydroxychloroquine with therapeutic regimens is in the intracellular cycle of the bacterium, which makes antimicrobials with high penetration into the intracellular environment preferable [62]. The ability of hydroxychloroquine to alkalize the pH of macrophages may be responsible for increasing the effect of antibiotics on intracellular bacteria, especially Brucella, providing conditions for antimicrobial efficacy [63–65].

In this review, the adverse event profile did not differ between the compared regimens. However, a great heterogeneity between the studies in relation to the safety outcome was observed, which is probably related to the lack of standardization in the definitions adopted to identify and characterize the events in terms of intensity and severity, in addition to the lack of systematization in monitoring the outcome. Another critical issue is that the adverse reaction profile and the other outcomes evaluated in this review are influenced by the participants’ characteristics and risk of relapse [66,67]. However, this information was not explored by the studies included in the previously published systematic reviews [12,15], nor was it detailed by the studies published in the last decade [16–20,55]. In any case, our results about adverse events need to be evaluated cautiously since not all studies reported this outcome systematically for all interventions assessed.

The choice of regimens for treating human brucellosis should consider efficacy and safety results. However, other aspects should be discussed in the decision-making process since the costs and preferences of users can significantly influence adherence and, consequently, the efficacy associated with each therapy [68,69] A previous study found that 60% of physicians prescribing treatment for brucellosis preferred a short-term triple therapy protocol [68]. In addition, convenience was one of the reasons for the therapy choice by these physicians, indicating the preference for the oral regimen (doxycycline + rifampicin), even though they were aware of the higher percentage of relapses compared to the other regimen [68]. Preference for the oral regimen was also identified in a study involving patients with brucellosis, with convenience identified as the main factor for this choice [69] the context of brucellosis as a neglected disease and without global initiatives to promote public education and improve a surveillance system [3,70], the present review reinforces findings and adds new evidence, representing a concrete opportunity to re-discuss treatment strategies and eradicate the disease in endemic areas. In particular, we highlight the need to consider the complexity of the health system and its capacity for administering parenteral drugs (intramuscular or intravenous), laboratory monitoring, and the availability of drugs as strategies to increase the effectiveness of treatments.

The greatest limitation of this review is the high risk of bias related to the included studies. In general, although the studies were randomized, there was no blinding of participants and researchers. Moreover, the measurement of outcomes was not presented in detail, and a lack of standardization in adopted definitions was observed, which incurs information bias. Finally, a few articles have mentioned previous registration of the study protocol to prevent selective reporting of the outcome. Future clinical trials should be concerned with meeting these criteria to allow the grouping and comparison of results in the search for more robust evidence to support therapeutic recommendations. Another important limitation refers to the scarcity of studies with direct comparisons for interventions of interest. Although NMA, in theory, tries to overcome the lack of direct comparisons between interventions, the approach has inherent limitations that increase uncertainty about results. In addition, the challenge of bringing together studies with participants’ details and with different clinical presentations of the disease limits our ability to identify the best therapeutic strategies for specific subgroups, as would be desirable.

This review adds relevant information to the scientific literature by presenting unprecedented comparisons between the interventions already studied for human brucellosis. Our results support the use of drugs previously indicated for treating human brucellosis, such as the combination of doxycycline and aminoglycosides. In addition, interventions related to treatment failure and others related to the possibility of superiority of triple therapy with hydroxychloroquine were identified, a result to be confirmed in future studies. This does not exclude the need for studies evaluating new combinations as they can be important for therapy to be achieved and adopted in different regions.

## Supporting information

S1 Table Search strategy. (DOCX)

S2 Table Studies excluded after full text reading. (DOCX)

S3 Table Definition of the outcomes. (DOCX)

S4 Table Certainty in the evidence for treatment overall therapy failure outcome. (DOCX)

S5 Table Certainty in the evidence for adverse events outcome. (DOCX)

S6 Table Risk of bias for overall therapy failure outcome. (DOCX)

S7 Table Risk of bias for adverse events outcome. (DOCX)

S8 Table Risk of bias for time to defervescence outcome. (DOCX)

S9 Table PRISMA Check list. (DOCX)
