# Supplementary material for: Efficacy and safety of therapeutic strategies for human brucellosis: A systematic review and network meta-analysis
Source: PLoS Negl Trop Dis. 2024 Mar 11;18(3):e0012010. doi: 10.1371/journal.pntd.0012010 (PMC10978012; doi:10.1371/journal.pntd.0012010)
Supplement: S1 Table — (DOCX) [file pntd.0012010.s001.docx]

**S1_Table_**Search strategy.

| **Database** | **Search strategy** | **Trials** |
| --- | --- | --- |
| MEDLINE (via Pubmed) | **Population:**  **#1** (Brucellosis[MeSH Terms]) OR (Brucellosis) OR (Brucelloses) OR (Malta Fever) OR (Fever, Malta) OR (Gibraltar Fever) OR (Fever, Gibraltar) OR (Rock Fever) OR (Fever, Rock) OR (Cyprus Fever) OR (Fever, Cyprus) OR (Brucella Infection) OR (Brucella Infections) OR (Infection, Brucella) OR (Undulant Fever) OR (Fever, Undulant) OR (Brucella abortus) OR (Brucella canis) OR (Brucella melitensis) OR (Brucella ovis) OR (Brucella suis) | 1472 |
|  | **Intevention:**  **#2** (tetracycline[MeSH Terms]) OR (doxycycline[MeSH Terms]) OR (gentamicin[MeSH Terms]) OR (ciprofloxacin[MeSH Terms]) OR (levofloxacin[MeSH Terms]) OR (ofloxacin[MeSH Terms]) OR (therapeutics[MeSH Terms]) OR(Tetracycline) OR (Tetrabid) OR (4-Epitetracycline) OR (4 Epitetracycline) OR (Topicycline) OR (Achromycin V) OR (Hostacyclin) OR (Tetracycline Hydrochloride) OR (Tetracycline Monohydrochloride) OR (Sustamycin) OR (Achromycin) OR (doxycycline) OR (Doxycycline Monohydrate) OR (Vibramycin) OR (Atridox) OR (Doxycycline Phosphate (1:1)) OR (BMY-28689) OR (BMY 28689) OR (BMY28689) OR (BU-3839T) OR (BU 3839T) OR (BU3839T) OR (Doryx) OR (Doxycycline Calcium Salt (1:2)) OR (Doxycycline Hyclate) OR (Doxycycline Hemiethanolate) OR (Doxycycline Monohydrochloride, 6-epimer) OR (Doxycycline Monohydrochloride, 6 epimer) OR (Doxycycline Monohydrochloride, Dihydrate) OR (Doxycycline Calcium) OR (2-Naphthacenecarboxamide, 4-(dimethylamino)-1,4,4a,5,5a,6,11,12a-octahydro-3,5,10,12,12a-pentahydroxy-6-methyl-1,11-dioxo-, (4S-(4alpha,4aalpha,5alpha,5aalpha,6alpha,12aalpha))-) OR (Alpha-6-Deoxyoxytetracycline) OR (Alpha 6 Deoxyoxytetracycline) OR (Doxycycline-Chinoin) OR (Doxycycline Chinoin) OR (Hydramycin) OR (Oracea) OR (Periostat) OR (Vibra-Tabs) OR (Vibra Tabs) OR (Vibramycin Novum) OR (Vibravenos) OR (minocycline) OR (Minox 50) OR (Aknemin) OR (Aknin-Mino) OR (Aknin Mino) OR (Aknosan) OR (Mynocine) OR (Apo-Minocycline) OR (Apo Minocycline) OR (Arestin) OR (Blemix) OR (Cyclomin) OR (Cyclops) OR (Dentomycin) OR (Dynacin) OR (Icht-Oral) OR (Icht Oral) OR (Klinomycin) OR (Lederderm) OR (Mestacine) OR (Minakne) OR (Mino-Wolff) OR (Mino Wolff) OR (Minocin) OR (Minocin MR) OR (Minoclir) OR (Minocycline Hydrochloride) OR (Hydrochloride, Minocycline) OR (Minocycline Monohydrochloride) OR (Monohydrochloride, Minocycline) OR (Minocycline, (4R-(4 alpha,4a beta,5a beta,12a beta))-Isomer) OR (Minolis) OR (Minomycin) OR (Minoplus) OR (Minotab) OR (Akamin) OR (Akne-Puren) OR (Akne Puren) OR (gentamicins) OR (Gentamycins) OR (Garamycin) OR (Gentacycol) OR (Gentamicin Sulfate) OR (Sulfate (Gentamicin) OR (Gentamicin Sulfate (USP)) OR (Gentavet) OR (Genticin) OR (G-Myticin) OR (G Myticin) OR (GMyticin) OR (Gentamicin) OR (Gentamycin) OR (streptomycin) OR (Streptomycine Panpharma) OR (Streptomycin Grünenthal) OR (Estreptomicina CEPA) OR (Strepto-Hefa) OR (Strepto Hefa) OR (Estreptomicina Clariana) OR (Estreptomicina Normon) OR (Strepto-Fatol) OR (Strepto Fatol) OR (Streptomycin Sulfate) OR (Streptomycin Sulfate (2:3) Salt) OR (Streptomycin Sulphate) OR (rifampicin) OR (Benemycin) OR (Rifampicin) OR (Rimactan) OR (Tubocin) OR (Rifadin) OR (Rimactane) OR (ciprofloxacin) OR (Ciprofloxacin Hydrochloride Anhydrous) OR (Anhydrous, Ciprofloxacin Hydrochloride) OR (Hydrochloride Anhydrous, Ciprofloxacin) OR (Cipro) OR (Ciprofloxacin Hydrochloride) OR (Hydrochloride, Ciprofloxacin) OR (Ciprofloxacin Monohydrochloride Monohydrate) OR (Monohydrate, Ciprofloxacin Monohydrochloride) OR (Monohydrochloride Monohydrate, Ciprofloxacin) OR (Bay-09867) OR (Bay 09867) OR (Bay09867) OR (Ciprinol) OR (levofloxacin) OR (Ofloxacin, (S)-Isomer) OR (Levofloxacin Anhydrous) OR (Anhydrous, Levofloxacin) OR (Quixin) OR (Levaquin) OR (ofloxacin) OR (Ofloxacine) OR (Tarivid) OR (ORF-28489) OR (ORF 28489) OR (ORF28489) OR (DR-3355) OR (DR 3355) OR (DR3355) OR (Hoe-280) OR (Hoe 280) OR (Hoe280) OR (Ofloxacin Hydrochloride) OR (Ru-43280) OR (Ru 43280) OR (Ru43280) OR (DL-8280) OR (DL 8280) OR (DL8280) OR (aminoglycosides) OR (macrolides) OR (macrolide) OR (Therapeutics) OR (Therapeutic) OR (Therapy) OR (Therapies) OR (Treatment) OR (Treatments) OR (Anti-Bacterial Agents) OR (Agents, Anti-Bacterial) OR (Anti Bacterial Agents) OR (Antibacterial Agents) OR (Agents, Antibacterial) OR (Antibacterial Agent) OR (Agent, Antibacterial) OR (Anti-Bacterial Compounds) OR (Anti Bacterial Compounds) OR (Compounds, Anti-Bacterial) OR (Anti-Bacterial Agent) OR (Agent, Anti-Bacterial) OR (Anti Bacterial Agent) OR (Anti-Bacterial Compound) OR (Anti Bacterial Compound) OR (Compound, Anti-Bacterial) OR (Bacteriocidal Agents) OR (Agents, Bacteriocidal) OR (Bacteriocidal Agent) OR (Agent, Bacteriocidal) OR (Bacteriocide) OR (Bacteriocides) OR (Antibiotics) OR (Antibiotic) |  |
|  | **Study design:**  **#3** ((("clinical"[Title/Abstract] AND "trial"[Title/Abstract]) OR "clinical trials as topic"[MeSH Terms] OR "clinical trial"[Publication Type] OR "random*"[Title/Abstract] OR "random allocation"[MeSH Terms] OR "therapeutic use"[MeSH Subheading]) AND "humans"[MeSH Terms]) NOT "animals"[MeSH Terms:noexp]    **#4**   #1 AND #2 AND #3 |  |
| EMBASE | **Population:**  **#1** 'brucellosis' OR 'brucella infection' OR 'brucella melitensis infection' OR 'malta fever' OR 'mediterranean fever (brucellosis)' OR 'brucellosis' OR 'infection by brucella' OR 'infection by brucella melitensis' OR 'infection due to brucella' OR 'infection due to brucella melitensis' OR 'melitococcosis' OR 'undulant fever' OR 'brucella' OR 'brucella abortus' OR 'brucella abortus' OR 'bacterium abortus' OR 'brucella abortus bang bacteria' OR 'brucella abortus sensitivity' OR 'brucella melitensis' OR 'brucella canis' OR 'brucella canis' OR 'brucella canidis' OR 'brucella ovis' OR 'brucella ovis' OR 'brucella suis' OR 'brucella melitensis biovar suis' OR 'brucella melitensisbv. suis' OR 'brucella suis' | 1727 |
|  | **Intervention:**  **#2** ‘Therapy’/syn OR 'doxycycline'/syn OR 'doxycycline' OR 'gentamicin'/syn OR 'gentamicin' OR 'rifampicin'/syn OR 'rifampicin' OR 'streptomycin'/syn OR 'streptomycin' OR 'ciprofloxacin'/syn OR 'ciprofloxacin' OR 'macrolide'/syn OR 'macrolide' OR 'aminoglycoside'/syn OR 'aminoglycoside' OR 'quinolone'/syn OR 'quinolone' |  |
|  | **Study design:**  **#3** (‘randomized controlled trial’/exp OR ‘clinical trial’/exp OR ‘comparative study’/exp OR ‘controlled study’/de OR ‘evaluation study’/de OR ‘human experiment’/exp OR random*:ab,ti OR control*:ab,ti OR ‘intervention study’:ab,ti OR ‘experimental study’:ab,ti OR trial:ab,ti OR trials:ab,ti OR compar*:ab,ti OR repeat*:ab,ti OR crossover:ab,ti OR ‘double blind’:ab,ti OR evaluat*:ab,ti OR ‘before and after’:ab,ti OR ‘interrupted time series’:ab,ti) NOT (‘animal’/exp NOT ‘human’/exp)    **#4**  #1 AND #2 AND #3 AND [humans]/lim AND [embase]/lim |  |
| Cochrane Library | **Population:**  **#1** MeSH descriptor: [Brucellosis] explode all trees    **Population:**  **#2** 'brucellosis' OR 'brucella infection' OR 'brucella melitensis infection' OR 'malta fever' OR 'mediterranean fever (brucellosis)' OR 'brucellosis' OR 'infection by brucella' OR 'infection by brucella melitensis' OR 'infection due to brucella' OR 'infection due to brucella melitensis' OR 'melitococcosis' OR 'undulant fever' OR 'brucella' OR 'brucella abortus' OR 'brucella abortus' OR 'bacterium abortus' OR 'brucella abortus bang bacteria' OR 'brucella abortus sensitivity' OR 'brucella melitensis' OR 'brucella canis' OR 'brucella canis' OR 'brucella canidis' OR 'brucella ovis' OR 'brucella ovis' OR 'brucella suis' OR 'brucella melitensis biovar suis' OR 'brucella melitensisbv. suis' OR 'brucella suis''brucellosis' OR 'brucella infection' OR 'brucella melitensis infection' OR 'malta fever' OR 'mediterranean fever (brucellosis)' OR 'brucellosis' OR 'infection by brucella' OR 'infection by brucella melitensis' OR 'infection due to brucella' OR 'infection due to brucella melitensis' OR 'melitococcosis' OR 'undulant fever' OR 'brucella' OR 'brucella abortus' OR 'brucella abortus' OR 'bacterium abortus' OR 'brucella abortus bang bacteria' OR 'brucella abortus sensitivity' OR 'brucella melitensis' OR 'brucella canis' OR 'brucella canis' OR 'brucella canidis' OR 'brucella ovis' OR 'brucella ovis' OR 'brucella suis' OR 'brucella melitensis biovar suis' OR 'brucella melitensisbv. suis' OR 'brucella suis'):ti,ab,kw | 84 |
|  | **Intervention:**  **#3** (Doxycycline OR Minocycline OR Rifampin OR Streptomycin OR Gentamicins OR Trimethoprim OR Sulfamethoxazole OR Quinolones OR Erythromycin OR Azithromycin OR Clarithromycin OR Macrolides OR Levofloxacin OR Ofloxacin OR Ciprofloxacin OR Chloramphenicol OR Tigecycline):ti,ab,kw    **#4** (#1 OR #2) AND #3 |  |
| BVS | **Population:**  **#1** ((mh:(brucelose)) OR (brucelose) OR (mh:(brucellosis)) OR (brucellosis) OR (mh:(brucelosis)) OR (brucelosis) OR (febre ondulante) OR (febre de malta) OR (infecçãopor brucella) OR (mh:(brucella)) OR (brucella) OR (mh:(brucella abortus)) OR (brucella abortus) OR (bacterium abortus) OR (mh:(brucella canis )) OR (brucella canis ) OR (mh:(brucella melitensis )) OR (brucella melitensis ) OR (micrococcus melitensis) OR (mh:(brucella ovis)) OR (brucella ovis) OR (mh:(brucella suis)) OR (brucella suis))    **Intervention:**  **#2** ((therapeutics OR therapeutic OR therapy OR therapies OR treatment OR treatments OR (anti-bacterial agents) OR (agents, anti-bacterial) OR (anti bacterial agents) OR (antibacterial agents) OR (agents, antibacterial) OR (antibacterial agent) OR (agent, antibacterial) OR (anti-bacterial compounds) OR (anti bacterial compounds) OR (compounds, anti-bacterial) OR (anti-bacterial agent) OR (agent, anti-bacterial) OR (anti bacterial agent) OR (anti-bacterial compound) OR (anti bacterial compound) OR (compound, anti-bacterial) OR (bacteriocidal agents) OR (agents, bacteriocidal) OR (bacteriocidal agent) OR (agent, bacteriocidal) OR (bacteriocide) OR (bacteriocides) OR (anti-mycobacterial agents) OR (agents, anti-mycobacterial) OR (anti mycobacterial agents) OR (anti-mycobacterial agent) OR (agent, anti-mycobacterial) OR (anti mycobacterial agent) OR (antimycobacterial agent) OR (agent, antimycobacterial) OR (antimycobacterial agents) OR (agents, antimycobacterial) OR (antibiotics) OR (antibiotic)) )    **Study design:**  **#3** (((mh:("Randomized Controlled Trials as Topic" OR "Controlled Clinical Trials as Topic" OR "Random Allocation" OR "Double-Blind Method" OR "Single-Blind Method" OR "Placebos" OR "Multicenter Studies as Topic" OR "Cross-Over Studies" OR "Pragmatic Clinical Trials as Topic") OR pt:("Randomized Controlled Trial" OR "Controlled Clinical Trial" OR "Multicenter Studies" OR "Pragmatic Clinical Trial") OR ti:(random* OR aleatori* OR placebo*) OR (ti:("clinical trial" OR "ensayoclinico" OR "ensaioclinico") AND tw:(control* OR random* OR aleatori* OR placebo*)) OR (ti:("cross-Over" OR multicenter OR multicentric*) AND ti:(study OR studies OR estud*)) OR ab:(randomi* OR aleatori* OR placebo*) OR (ab:("clinical trial" OR "ensayoclinico" OR "ensaioclinico") AND tw:(control* OR random* OR aleatori* OR placebo*)) OR (ab:("cross-Over" OR multicenter OR multicentric*) AND ab:(study OR studies OR estud*)) OR (tw:(simple* OR singl* OR duplo* OR doble* OR doubl* OR trebl* OR tripl*) AND tw:(cego OR ciego OR blind OR mask OR dumm*))) AND NOT ((mh:"animals" AND NOT mh:"humans") OR mh:"Retrospective Studies")))    **Bases available after withdrawing MEDLINE in the VHL filter:**  **#4** (db:("LILACS" OR "ARGMSAL" OR "BINACIS"))    **#5**  #1 AND #2 AND #3 AND #4 | 4 |
| **Total** | | **3.287** |
