# Supplementary material for: Efficacy and safety of therapeutic strategies for human brucellosis: A systematic review and network meta-analysis
Source: PLoS Negl Trop Dis. 2024 Mar 11;18(3):e0012010. doi: 10.1371/journal.pntd.0012010 (PMC10978012; doi:10.1371/journal.pntd.0012010)
Supplement: S2 Table — (DOCX) [file pntd.0012010.s002.docx]

**S2_Table_**Studies excluded after full text reading.

| Author, year | Title | Reason for exclusion |
| --- | --- | --- |
| Ahmadvand, 2021 | The Effectiveness of Vitamin A on the Symptoms of Brucellosis | Wrong outcome |
| Al Anazi, 2012 | The effect of levamisole combined with standard treatment vs. standard treatment on the functions of polymorphonulcear cells and monocytes in patients with brucellosis | Wrong outcome |
| Alikhani, 2007 | A new therapeutic management in acute uncomplicated brucellosis: Comparison between three month-ofloxacin and doxycycline-based regimens | Language (Persian) |
| Alp, 2006 | Doxycycline plus streptomycin versus ciprofloxacin plus rifampicin in spinal brucellosis | Wrong study design and wrong population (secondary manifestation of brucellosis) |
| Aminov, 2020 | Improvement of treatment protocols of pain syndrome in patients with chronic brucellosis | Wrong outcome |
| Ariza, 1982 | Comparative trial of rifampicin-trimethoprim versus tetracycline-streptomycin in acute brucellosis | Not retrieved |
| Ariza, 1985 | Comparative trial of rifampin-doxycycline versus tetracycline-streptomycin in the therapy of human brucellosis. | The outcome cannot be assessed separately for interventions. |
| Barba, 1968 | Controlled research on the activity of a new antibiotic-antiphlogistic drug association in the treatment of typhoid and brucellosis. | Language (Italian) |
| Bertrand, 1979 | The treatment of brucellosis using rifampicine (author's transl). | Language (French) |
| Burns, 1955 | Therapeutic experiences with erythromycin alone and in combination with streptomycin in the treatment of brucellosis. | Not retrieved |
| Carrington, 1982 | Comparison of three drugs regimens in the treatment of brucellosis | Not retrieved |
| Cisneros, 1990 | Multicenter prospective study of treatment of Brucella melitensis brucellosis with doxycycline for 6 weeks plus streptomycin for 2 weeks. | Wrong study design (no comparator group) |
| Didgar, 2012 | Comparison between efficacy of ciprofioxacin-doxycycline and rifampin-doxycycline regimens in treatment of brucellosis | Language (Persian) |
| Duasenova, 2002 | Ciprofloxacin in the treatment of patients with brucellosis. | Language (Russian) |
| Fiaccadori, 1972 | Comparative study of the use of tetracycline in low dosage in the treatment of brucellosis | Language (Italian) |
| Goktas, 1992 | Comparison of streptomycin-tetracycline versus streptomycin-doxycycline in the treatment of brucellosis. The relationship between delay in therapy and its becoming chronic | Not retrieved |
| Hashemi, 2011 | Comparison of doxycycline-streptomycin, Doxycyclinerifampin and ofloxacin-rifampin in the treatment of human brucellosis | Duplicate (abstract with results published in full paper) |
| Irmak, 2003 | The effect of levamisole combined with the classical treatment in chronic brucellosis. | Wrong outcome |
| Kosmidis,1982 | The combination rifampicin/doxycyclin in brucellosis is better than the WHO regime | Not retrieved |
| Lang, 1990 | Failure of prolonged treatment with ciprofloxacin in acute infections due to Brucella melitensis | Study population less than 10 patients per arm |
| Lang, 1992 | Failure of ceftriaxone in the treatment of acute brucellosis. |  |
| Lyapina, 2015 | Inflammatory diseases of scrotal organs in patients with brucellosis:  Improvement of therapy. | Wrong outcome |
| McDevitt. 1970 | Ampicillin in the treatment of brucellosis. A controlled therapeutic trial. | Wrong outcome |
| Mukovozova, 1986 | Comparative effectiveness of levamisole and vaccine in the complex treatment of patients with brucellosis. | Language (Russian) |
| Mukovozova, 1986 | Effectiveness of levamisole in the complex treatment of patients with active forms of brucellosis. | Language (Russian) |
| Mukovozova, 1987 | Efficacy of various methods of therapy of chronic brucellosis. | Language (Russian) |
| Mukovozova, 1988 | Effect of levamisole on the cellular and humoral immunity indices in patients with chronic brucellosis. | Language (Russian) |
| Roushan, 2009 | Optimal duration of gentamicin containing regimen for the treatment of uncomplicated brucellosis | Duplicate (abstract with results published in full paper) |
| Solera, 1992 | Multicentre comparative therapeutic trial of rifampicin and doxycycline versus streptomycin and doxycycline in human brucellosis. Primary evaluation results | Not retrieved |
| Solera, 2001 | Azithromycin and gentamicin therapy for the treatment of humans with brucellosis. | Wrong study design (no comparator group) |
| Vargas,1980 | Treatment of acute brucellosis with cotrimoxazole, doxicyclin and streptomycin.  A comparative study (author's transl). | Not retrieved |
| Vrioni, 2014 | Administration of a triple versus a standard double antimicrobial regimen for human brucellosis more efficiently eliminates bacterial DNA load. | Wrong study design and wrong outcome |

**REFERENCE**

1. Ahmadvand,N., Zarinfar, N., Soofian, M. The Effectiveness of Vitamin A on the Symptoms of Brucellosis. Journal of Babol University of Medical Sciences. 2021;23: 252–258. doi:10.22088/jbums.23.1.252

2. Al Anazi A., Al Aska, A., Al Tuwaijri, A., Al-Orainey, I., Al-Hedaithy, M., Al Majid, F., et al. The effect of levamisole combined with standard treatment vs. standard treatment on the functions of polymorphonulcear cells and monocytes in patients with brucellosis. Clinical Microbiology and Infection. 2012;18: 62. doi:10.1111/j.1469-0691.2012.03801.x

3. Alikhani, A., Heidarzadeh, A. A new therapeutic management in acute uncomplicated brucellosis: Comparison between three month-ofloxacin and doxycycline-based regimens. Journal of Isfahan Medical School. 2007;25. Available: https://www.embase.com/search/results?subaction=viewrecord&id=L621705919&from=export

4. Alp, E., Koc, R., Durak, A., Yildiz, O., Aygen, B., Sumerkan, B., et al. Doxycycline plus streptomycin versus ciprofloxacin plus rifampicin in spinal brucellosis. BMC Infect Dis. 2006;6: 72. doi:10.1186/1471-2334-6-72

5. Aminov, Z., Khakimova, S., Davlatov, S. Improvement of treatment protocols of pain syndrome in patients with chronic brucellosis. European Journal of Molecular and Clinical Medicine. 2020;7: 2540–2545. Available: https://www.embase.com/search/results?subaction=viewrecord&id=L2010454919&from=export

6. Ariza, J., Gudiol, F., Fernandez, P. Comparative trial of rifampicin-trimethoprim versus tetracycline-streptomycin in acute brucellosis. Chemioterapia. 1982;1: No. 442-No. 442. Available: https://www.embase.com/search/results?subaction=viewrecord&id=L13140880&from=export

7. Ariza, J., Gudiol, F., Pallarés, R., Rufí, G., Fernández-Viladrich, P. Comparative trial of rifampin-doxycycline versus tetracycline-streptomycin in the  therapy of human brucellosis. Antimicrob Agents Chemother. 1985;28: 548–551. doi:10.1128/AAC.28.4.548

8. Barba, G., Frigerio, G., Bruno, F. [Controlled research on the activity of a new antibiotic-antiphlogistic drug  association in the treatment of typhoid and brucellosis]. Minerva Med. 1968;59: 1528–1534.

9. Bertrand, A., Roux, J., Janbon, F., Jourdan, J., Jonquet, O. [The treatment of brucellosis using rifampicine (author’s transl)]. Nouv Presse Med. 1979;8: 3635–3639.

10. Burns, T., Alfi, O., Badran, A., Pfischner, W., Killough, J., Farid, Z. Therapeutic experiences with erythromycin alone and in combination with  streptomycin in the treatment of brucellosis. J Med Liban. 1955;8: 375–383.

11. Carrington da Costa, R., Corte Real, R., Pereira, A. Comparison of three drugs regimens in the treatment of brucellosis. Chemioterapia. 1982;1: No. 219-No. 219. Available: https://www.embase.com/search/results?subaction=viewrecord&id=L13111480&from=export

12. Cisneros, J., Viciana, P., Colmenero, J., Pachón, J., Martinez, C., Alarcón, A. Multicenter prospective study of treatment of Brucella melitensis brucellosis  with doxycycline for 6 weeks plus streptomycin for 2 weeks. Antimicrob Agents Chemother. 1990;34: 881–883. doi:10.1128/AAC.34.5.881

13. Didgar, F., Sarmadian, H., Zarin Far, N., Rafiee, M., Choghae, M. Comparison between efficacy of ciprofioxacin-doxycycline and rifampin-doxycycline regimens in treatment of brucellosis. Journal of zanjan university of medical sciences and health services. 2012;20: 2.

14. Duĭsenova, A., Kurmanova, K., Kurmanova, G. Ciprofloxacin in the treatment of patients with brucellosis. Antibiot Khimioter. 2002;47: 3–7.

15. Fiaccadori, F., Camilloni, R., Ghinelli, F., Pizzigoni, G. Comparative study of the use of tetracycline in low dosage in the treatment of brucellosis. G Clin Med. 1972;53: 290–299. Available: https://www.cochranelibrary.com/central/doi/10.1002/central/CN-00008269/full

16. Goktas, P. Comparison of streptomycin-tetracycline versus streptomycin-doxycycline in the treatment of brucellosis. The relationship between delay in therapy and its becoming chronic. Curr Ther Res Clin Exp. 1992;51: 553–561. Available: https://www.embase.com/search/results?subaction=viewrecord&id=L22129275&from=export

17. Hashemi, S., Gachkar, L., Keramat, F., Mamani, M., Hajilooi, M., Janbakhsh, A., et al. Comparison of doxycycline-streptomycin, Doxycyclinerifampin and ofloxacin-rifampin in the treatment of human brucellosis. Clinical Microbiology and Infection. 2011;17: S441–S442. doi:10.1111/j.1469-0691.2011.03558.x

18. Irmak, H., Buzgan, T., Karahocagil, M., Evirgen, O., Akdeniz, H., Demiröz, A. The effect of levamisole combined with the classical treatment in chronic  brucellosis. Tohoku J Exp Med. 2003;201: 221–228. doi:10.1620/tjem.201.221

19. Kosmidis, J., Karagounis, A., Tselentis, J., Daikos, G. The combination rifampicin/doxycyclin in brucellosis is better than the WHO regime. Chemioterapia. 1982;1: No. 222-No. 222. Available: https://www.embase.com/search/results?subaction=viewrecord&id=L13111483&from=export

20. Lang, R., Raz, R., Sacks, T., Shapiro, M. Failure of prolonged treatment with ciprofloxacin in acute infections due to Brucella melitensis. Journal of antimicrobial chemotherapy. 1990;26: 841–846. doi:10.1093/jac/26.6.841

21. Lyapina, E., Shuldyakov, A., Evdokimov, A., Glybochko, P. [Inflammatory diseases of scrotal organs in patients with brucellosis:  Improvement of therapy]. Ter Arkh. 2015;87: 56–61. doi:10.17116/terarkh2015871156-61

22. McDevitt, D. Ampicillin in the treatment of brucellosis. A controlled therapeutic trial. Br J Ind Med. 1970;27: 67–71. doi:10.1136/oem.27.1.67

23. Mukovozova, L. [Comparative effectiveness of levamisole and vaccine in the complex treatment of  patients with brucellosis]. Ter Arkh. 1986;58: 43–45.

24. Mukovozova, L. [Effectiveness of levamisole in the complex treatment of patients with active  forms of brucellosis]. Klin Med (Mosk). 1986;64: 47–51.

25. Mukovozova, L. [Efficacy of various methods of therapy of chronic brucellosis]. Ter Arkh. 1987;59: 93–96.

26. Mukovozova, L. [Effect of levamisole on the cellular and humoral immunity indices in patients  with chronic brucellosis]. Zh Mikrobiol Epidemiol Immunobiol. 1988; 45–49.

27. Roushan, M., Amiri, M., Janmohammadi, N., Javanian, M., Baiani, M. Optimal duration of gentamicin containing regimen for the treatment of uncomplicated brucellosis. Clinical Microbiology and Infection. 2009;15: S47–S48. doi:10.1111/j.1469-0691.2009.02857.x

28. Solera, J., Paulino, J., Rodrígeuz-Zapata, M., Geijó, P., Medrano, F., Jiménez-Zorzo, F., et al. Multicentre comparative therapeutic trial of rifampicin and doxycycline versus streptomycin and doxycycline in human brucellosis. Primary evaluation results. Revista española de reumatología. 1992;19: 219. Available: https://www.cochranelibrary.com/central/doi/10.1002/central/CN-00309343/full

29. Solera, J., Beato, J., Martínez-Alfaro, E., Segura, J., de Tomas, E. Azithromycin and gentamicin therapy for the treatment of humans with brucellosis. Clin Infect Dis. 2001;32: 506–509. doi:10.1086/318503

30. Vargas, V., Pedreira, J., Clotet, B., Juste, C., Guardia, J., Bacardi, R. [Treatment of acute brucellosis with cotrimoxazole, doxicyclin and streptomycin.  A comparative study (author’s transl)]. Med Clin (Barc). 1980;75: 418–420.

31. Vrioni, G., Bourdakis, A., Pappas, G., Pitiriga, V., Mavrouli, M., Pournaras, S., et al. Administration of a triple versus a standard double antimicrobial regimen for  human brucellosis more efficiently eliminates bacterial DNA load. Antimicrob Agents Chemother. 2014;58: 7541–7544. doi:10.1128/AAC.03841-14
