# Supplementary material for: Efficacy and safety of therapeutic strategies for human brucellosis: A systematic review and network meta-analysis
Source: PLoS Negl Trop Dis. 2024 Mar 11;18(3):e0012010. doi: 10.1371/journal.pntd.0012010 (PMC10978012; doi:10.1371/journal.pntd.0012010)
Supplement: S3 Table — (DOCX) [file pntd.0012010.s003.docx]

**S3_Table_**Definition of the outcomes.

| Year, Author | Definition of the outcomes |
| --- | --- |
| 1973, Feiz | **Relapse:** reappearance of symptoms of brucellosis or bacteriaemia persisted after the treatment course was completed.  **Clinical response:** improvement of clinical symptoms (arthritis, headache, joint pains, sweating and neuralgia) and the temperature was normal. |
| 1982, Buzon | Not Reported. |
| 1985, Ariza | **Relapse:** reappearance of signs or symptoms of the disease or new positive cultures of blood after therapy.  **Defervescence:** number of days from start of therapy until the patient became afebrile. |
| 1987, Rodriguez Zapata | **Relapse:** initial disappearance of symptoms these reappeared at any time during the follow-up period.  **Failure:** treatment had to be stopped during its time of application due to overt signs of lack of efficacy and/or occurrence of sided effects clearly attributable to the treatment, and if clinical symptoms of the disease persisted in spite of the continuation of the treatment up to the pre-established duration.  **Cure:** all symptoms and signs of the disease disappeared completely and there was no relapse during the 12 months of the follow-up period. |
| 1989, Acocella | **Relapse**: when symptoms reappeared at any time during the follow up period.  **Failure:** when the treatment had to be stopped prematurely because of overt signs of lack of efficacy and/or occurrence of side effects clearly attributable to the treatment.  **Unsuccessful:** when clinical symptoms of the disease persisted in spite of the continuation of the treatment up to the pre-established duration.  **Cure:** when all symptoms and signs of the disease disappeared completely and there was no relapse during the 12 months follow up period. |
| 1989, Colmenero | **Relapse:** recurrence of symptoms after the termination of treatment.  **Therapeutic failures:** persistence of signs or symptoms of the disease after 15 days of treatment. |
| 1991, Solera | **Relapse:** reappearance of signs or symptoms of the disease and/or new positivity of blood cultures after completion of treatment.  **Therapeutic failure:** persistence of symptoms or symptoms of pain 21 days after the start of the treatment, or time in which the treatment was able to control the fever. |
| 1992, Ariza | **Relapse:** Reappearance of symptoms or sings of the disease or new positive blood cultures during 12 months after therapy.  **Therapeutic failure:** symptoms or signs of the disease persisted at the end of treatment.  **Defervescence:** the number of days from the time the patient started treatment to the time the patient became afebrile. |
| 1993, Akova | **Relapse:** reappearance of symptoms or signs or new positive blood or bone marrow cultures during the 12 months after therapy.  **Therapeutic failure:** persistence of symptoms or signs of the disease, or both, at the end of therapy.  **Time to** **defervescence:** the number of days elapsed from the start of therapy until the patient became afebrile. |
| 1993, Montejo | **Relapse:** indicative clinical picture reappeared and/or the blood cultures were positive after the end of the course of treatment.  **Therapeutic failure:** persistence of the clinical picture associated with the disease 2 weeks after antimicrobial treatment had begun. |
| 1994, Colmenero | **Relapse:** reappearance of symptoms once the treatment had finished.  **Therapeutic failure:** persistence of symptoms after the third week of treatment. |
| 1995, Solera | **Relapse:** reappearance of symptoms or signs of the disease or new positive blood cultures during 12 months after therapy.  **Therapeutic failure:** symptoms or signs of the disease persisting after 4 weeks of treatment. |
| 1996, Kalo | **Relapse:** persistence of symptoms after 7 days of the treatment, and new blood samples for cultures were collected from patients suspected of having a relapse. |
| 1999, Agalar | **Relapse:** reappearance of symptoms and signs of the disease accompanied by increasing values of the serologic tests during the 12-month follow-up period after treatment was stopped. |
| 2002, Saltoglu | **Relapse:** reappearance of signs or symptoms of the disease throughout the follow-up or by increasing values of STA test result, or both. |
| 2004, Solera | **Relapse:** reappearance of symptoms or signs of the disease (as assessed by the patient’s physician) or a new positive result of blood culture within the 12-month period after the end therapy. |
| 2004, Karabay | **Relapse:** reappearance of symptoms and sign of the disease accompanied by increasing titers of the serological tests and/or a positive culture during the follow-up period after treatment was stopped. |
| 2004, Roushan | **Relapse:** indicative clinical picture reappeared and reduced titers of STAT, and 2 ME and brucella specific IgG titers after completion of therapy, increased again.  **Therapeutic failure:** symptoms or signs of the disease that persisted at the end of treatment.  **Failure of treatment:** therapeutic failure, adverse effects of medication or refused to have follow up. |
| 2005, Ersoy | **Relapse:** reappearance of clinical symptoms with laboratory findings. |
| 2006, Roushan | **Relapse:** when clinical symptoms and signs of brucellosis reappeared and a reduced titer of STAT or 2ME increased after completion of therapy.  **Therapeutic failure:** persistence of the clinical symptoms of disease after completion of treatment or discontinuation of treatment due to serious adverse effects associated with ≥1 of the drugs. |
| 2007, Alavi | **Relapse:** when the indicative clinical picture reappeared and reduced titers of Wright and 2 ME after completion of therapy, increased again.  **Therapeutic failure:** persistence of fever, sweating or back pain at the end of treatment.  **Clinical response:** was defined by subsiding fever, back pain and sweating. |
| 2007, Ranjbar | **Relapse:** reappearance of symptoms or signs of the disease or new positive blood cultures after therapy.  **Therapeutic failure:** persistence of symptoms or signs of the disease at the end of treatment. |
| 2009, Keramat | **Relapse:** reappearance of symptoms and signs of the disease accompanied by increasing titres of the serological tests during the follow-up period.  **Therapeutic failure** if symptoms and signs of the disease persisted or had increased at the end of eight to 12 weeks of therapy.  **Response to therapy:** improvement of symptoms and signs at the end of eight to 12 weeks of therapy (the first therapeutic course). |
| 2009, Sarmadian | **Relapse:** reappearance of symptoms after completing the treatment and during the follow up period of 6 months. |
| 2010, Roushan | **Relapse:** clinical symptoms and signs of brucellosis reappeared and reduced titres of STA or 2ME increased again, or the Brucella species was isolated from blood culture during the follow-up period.  **Therapeutic failure:** persistence or worsening of the symptoms or signs of the disease at the end of treatment, as judged clinically. |
| 2012, Hashemi | **Relapse:** the reappearance of symptoms and signs accompanied by a 2-ME titer ≥1/80 during the follow-up period.  **Therapeutic Failure:** persistence of symptoms and signs at the end of 6 weeks of therapy.  **Clinical response:**  clinical improvement of primary signs (objective) and symptoms (subjective) of disease at the end of treatment recorded apart for each patient. |
| 2014, Sofian | **Relapse:** reappearance of brucellosis signs and symptoms and a rise in antibody titers at the end of treatment or during the follow-up period.  **Clinical response:** complete relief of fever and symptoms. |
| 2016, Hasanain | **Relapse:** recurrence of the clinical manifestations with a single positive antibody titer within six months after ending therapy.  **Therapeutic failure:** persistence of the clinical manifestations of brucellosis at end of treatment (after six weeks of treatment). |
| 2018, Majzoobi | **Relapse:** initial clinical response, then recurrence of disease symptoms accompanied by increase in 2ME titer.  **Treatment failure:** persistence or worsening of clinical symptoms, and lack of reduction, or increase, of 2ME titer. |
| 2020, Karami | **Relapse:** recurrence of the clinical symptoms of brucellosis as confirmed by the serological tests (increased 2ME and Wright compared to the post-treatment serology). |
| 2022, Majzoobi | **Relapse:** recurrence of clinical symptoms with increase in 2ME titer toward the end of treatment was defined as relapse. |

**REFERENCE**

1. Feiz, J., Sabbaghian, H. & Sohrabi, F. A comparative study of therapeutic agents used for treatment of acute brucellosis. *Br J Clin Pract* **27**, 410–413 (1973).

2. Buzon, L., Bouza, E. & Rodriguez, M. Treatment of brucellosis with rifampicin+tetracycline vs TMP/SMZ. A prospective and randomized study. *Chemioterapia* **1**, No. 221-No. 221 (1982).

3. Ariza, J., Gudiol, F., Pallarés, R., Rufí, G. & Fernández-Viladrich, P. Comparative trial of co-trimoxazole versus tetracycline-streptomycin in treating human brucellosis. *Journal of infectious diseases* **152**, 1358–1359 (1985).

4. Rodriguez Zapata, M., Gamo Herranz, A. & De La Morena Fernández, J. Comparative study of two regimens in the treatment of brucellosis. *Chemioterapia* **6**, 360–362 (1987).

5. Acocella, G. *et al.* Comparison of three different regimens in the treatment of acute brucellosis: a multicenter multinational study. *J Antimicrob Chemother* **23**, 433–439 (1989).

6. Colmenero Castillo, J., Hernandez Marquez, S., Reguera Iglesias, J., Cabrera Franquelo, F., Rius Diaz, F., & Alonso, A *.* Comparative trial of doxycycline plus streptomycin versus doxycycline plus rifampin for the therapy of human brucellosis. *Chemotherapy* **35**, 146–152 (1989).

7. Solera, J., Medrano, F., Rodríguez, M., Geijo, P. & Paulino, J. [A comparative therapeutic and multicenter trial of rifampicin and doxycycline versus streptomycin and doxycycline in human brucellosis]. *Med Clin (Barc)* **96**, 649–653 (1991).

8. Ariza, J. *et al.* Treatment of human brucellosis with doxycycline plus rifampin or doxycycline plus streptomycin. A randomized, double-blind study. *Ann Intern Med* **117**, 25–30 (1992).

9. Akova, M., Uzun, O., Akalin, H. E., Hayran, M., Unal, S., & Gür, D*.* Quinolones in treatment of human brucellosis: comparative trial of ofloxacin-rifampin versus doxycycline-rifampin. *Antimicrob Agents Chemother* **37**, 1831–1834 (1993).

10. Montejo, J., Alberola, I., Glez-Zarate, P., Alvarez, A., Alonso, J., Canovas, A., & Aguirre, C. Open, randomized therapeutic trial of six antimicrobial regimens in the treatment of human brucellosis. *Clinical infectious diseases* **16**, 671–676 (1993).

11. Colmenero, J., Porras, J., Cárdenas, A., Ocón, P., Reguera, J., Delgado, M., & Sedeño, J*.* Evaluation of the Chromotitre EIA test for the diagnosis of human brucellosis. *Enferm Infecc Microbiol Clin* **12**, 60–65 (1994).

12. Solera, J. *et al.* Doxycycline-rifampin versus doxycycline-streptomycin in treatment of human brucellosis due to Brucella melitensis. *Antimicrob Agents Chemother* **39**, 2061–2067 (1995).

13. Kalo, T., Novi, S., Nushi, A. & Dedja, S. Ciprofloxacin plus doxycycline versus rifampicin plus doxycycline in the treatment of acute brucellosis. *Med Mal Infect* **26**, 587–589 (1996).

14. Agalar, C., Usubutun, S. & Turkyilmaz, R. Ciprofloxacin and rifampicin versus doxycycline and rifampicin in the treatment of brucellosis. *European journal of clinical microbiology & infectious diseases* **18**, 535–538 (1999).

15. Saltoglu, N., Tasova, Y., Inal, A. S., Seki, T. & Aksu, H. S. Efficacy of rifampicin plus doxycycline versus rifampicin plus quinolone in the treatment of brucellosis. *Saudi Med J* **23**, 921–924 (2002).

16. Solera, J. *et al.* A randomized, double-blind study to assess the optimal duration of doxycycline treatment for human brucellosis. *Clinical infectious diseases* **39**, 1776–1782 (2004).

17. Karabay, O., Sencan, I., Kayas, D. & Sahin, I. Ofloxacin plus rifampicin versus doxycycline plus rifampicin in the treatment of brucellosis: a randomized clinical trial [ISRCTN11871179]. *BMC Infect Dis* **4**, 18–18 (2004).

18. Roushan, M., Gangi, S. M. E. & Ahmadi, S. A. A. Comparison of the efficacy of two months of treatment with co-trimoxazole plus doxycycline vs. co-trimoxazole plus rifampin in brucellosis. *Swiss Med Wkly* **134**, 564–568 (2004).

19. Ersoy, Y., Sonmez, E., Tevfik, M. R. & But, A. D. Comparison of three different combination therapies in the treatment of human brucellosis. *Trop Doct* **35**, 210–212 (2005).

20. Roushan, M., Mohraz, M., Janmohammadi, N. & Hajiahmadi, M. Efficacy of cotrimoxazole and rifampin for 6 or 8 weeks of therapy in childhood brucellosis. *Pediatr Infect Dis J* **25**, 544–545 (2006).

21. Alavi, S. & Rajabzadeh, A. Comparison of two chemotherapy regimen: doxycycline-rifampicin and doxycycline cotrimoxazol in the brucellosis patients Ahvaz, Iran, 2004-2006. *Pak J Med Sci* **23**, 889–892 (2007).

22. Ranjbar, M. *et al.* Comparison between doxycycline-rifampin-amikacin and doxycycline-rifampin regimens in the treatment of brucellosis. *International Journal of Infectious Diseases* **11**, 152–156 (2007).

23. Keramat, F., Ranjbar, M., Mamani, M., Hashemi, S. H. & Zeraati, F. A comparative trial of three therapeutic regimens: ciprofloxacin-rifampin, ciprofloxacin-doxycycline and doxycycline-rifampin in the treatment of brucellosis. *Trop Doct* **39**, 207–210 (2009).

24. Sarmadian, H., Didgar, F., Sufian, M., Zarinfar, N. & Salehi, F. Comparison Between Efficacy of Cipofoxacin Doxycycline and Rifampin - Doxycycline Regimens in Treatment and Relapse of Brucellosis. *Tropical medicine & international health* **14**, 209–209 (2009).

25. Roushan, M., Amiri, M., Janmohammadi, N., Hadad, M., Javanian, M., Baiani, M., & Bijani, A. Comparison of the efficacy of gentamicin for 5 days plus doxycycline for 8 weeks versus streptomycin for 2 weeks plus doxycycline for 45 days in the treatment of human brucellosis: a randomized clinical trial. *J Antimicrob Chemother* **65**, 1028–1035 (2010).

26. Hashemi, S. *et al.* Comparison of doxycycline-streptomycin, doxycycline-rifampin, and ofloxacin-rifampin in the treatment of brucellosis: a randomized clinical trial. *International journal of infectious diseases* **16**, e247-51 (2012).

27. Sofian, M. *et al.* Comparison of two durations of triple-drug therapy in patients with uncomplicated brucellosis: A randomized controlled trial. *Scand J Infect Dis* **46**, 573–577 (2014).

28. Hasanain, A., Mahdy, R., Mohamed, A. & Ali, M. A randomized, comparative study of dual therapy (doxycycline-rifampin) versus triple therapy (doxycycline-rifampin-levofloxacin) for treating acute/subacute brucellosis. *Brazilian Journal of Infectious Diseases* **20**, 250–254 (2016).

29. Majzoobi, M. *et al.* Effect of hydroxychloroquine on treatment and recurrence of acute brucellosis: a single-blind, randomized clinical trial. *Int J Antimicrob Agents* **51**, 365–369 (2018).

30. Karami, A., Mobaien, A., Jozpanahi, M., Moghtader-Mojdehi, A. & Javaheri, M. Effect of 8-week and 12-week triple therapy (doxycycline, rifampicin, and gentamicin) on brucellosis: A comparative study. *Journal of Acute Disease* **9**, 161–165 (2020).

31. Majzoobi, M. M., Hashmi, S. H., Emami, K. & Soltanian, A. R. Combination of doxycycline, streptomycin and hydroxychloroquine for short-course treatment of brucellosis: a single-blind randomized clinical trial. *Infection* **50**, 1267–1271 (2022).
