# Supplementary material for: Efficacy and safety of therapeutic strategies for human brucellosis: A systematic review and network meta-analysis
Source: PLoS Negl Trop Dis. 2024 Mar 11;18(3):e0012010. doi: 10.1371/journal.pntd.0012010 (PMC10978012; doi:10.1371/journal.pntd.0012010)
Supplement: S4 Table — (DOCX) [file pntd.0012010.s004.docx]

**S4_Table_**Certainty in the Evidence for overall therapy failure outcome.

| **Comparison** | **Number of studies** | **Within-study bias** | **Reporting bias** | **Indirectness** | **Imprecision** | **Heterogeneity** | **Incoherence** | **Confidence rating** |
| --- | --- | --- | --- | --- | --- | --- | --- | --- |
| DX+GT:DX+GT(30 days) | 1 | Major concerns | Low risk | Some concerns | Major concerns | No concerns | Major concerns | Very low |
| DX+GT:DX+STP | 2 | Major concerns | Low risk | No concerns | Major concerns | No concerns | Major concerns | Very low |
| DX+RF:DX+STP | 10 | Major concerns | Low risk | No concerns | No concerns | Major concerns | No concerns | Very low |
| DX+STP:DX+STP+HC (42days) | 1 | Some concerns | Low risk | No concerns | No concerns | Some concerns | Major concerns | Very low |
| DX+STP+HC (28days):DX+STP+HC (42days) | 1 | Major concerns | Low risk | Some concerns | Major concerns | No concerns | Major concerns | Very low |
| DX+GT:DX+RF | 0 | Major concerns | Low risk | No concerns | No concerns | Major concerns | Major concerns | Very low |
| DX+GT:DX+STP+HC (28days) | 0 | Major concerns | Low risk | No concerns | Major concerns | No concerns | Major concerns | Very low |
| DX+GT:DX+STP+HC (42days) | 0 | Major concerns | Low risk | No concerns | Major concerns | No concerns | Major concerns | Very low |
| DX+GT(30 days):DX+RF | 0 | Major concerns | Low risk | No concerns | Major concerns | No concerns | Major concerns | Very low |
| DX+GT(30 days):DX+STP | 0 | Major concerns | Low risk | Some concerns | Major concerns | No concerns | Major concerns | Very low |
| DX+GT(30 days):DX+STP+HC (28days) | 0 | Major concerns | Low risk | Some concerns | Major concerns | No concerns | Major concerns | Very low |
| DX+GT(30 days):DX+STP+HC (42days) | 0 | Major concerns | Low risk | No concerns | Major concerns | No concerns | Major concerns | Very low |
| DX+RF:DX+STP+HC (28days) | 0 | Major concerns | Low risk | No concerns | No concerns | Some concerns | Major concerns | Very low |
| DX+RF:DX+STP+HC (42days) | 0 | Major concerns | Low risk | No concerns | No concerns | No concerns | Major concerns | Very low |
| DX+STP:DX+STP+HC (28days) | 0 | Major concerns | Low risk | Some concerns | Major concerns | No concerns | Major concerns | Very low |

DX: Doxycycline; RF: Rifampicin; STP: Streptomycin; GT: gentamicin; HC: hydroxychloroquine
