# Supplementary material for: Efficacy and safety of therapeutic strategies for human brucellosis: A systematic review and network meta-analysis
Source: PLoS Negl Trop Dis. 2024 Mar 11;18(3):e0012010. doi: 10.1371/journal.pntd.0012010 (PMC10978012; doi:10.1371/journal.pntd.0012010)
Supplement: S6 Table — (DOCX) [file pntd.0012010.s006.docx]

**S6_Table_**Risk of bias for overall therapy failure outcome.

| **Year, Author** | **Domain 1** | **Domain 2** | **Domain 3** | **Domain 4** | **Domain 5** | **Risk of bias** |
| --- | --- | --- | --- | --- | --- | --- |
| 1985, Ariza | High | Low | Low | High | High | High |
| 1987, Rodriguez Zapata | High | Low | Low | High | High | High |
| 1989, Acocella | Low | Low | Low | High | High | High |
| 1989, Colmenero | High | Low | Low | High | High | High |
| 1991, Solera | High | Low | Low | High | High | High |
| 1992, Ariza | Low | Low | Low | High | High | High |
| 1993, Akova | High | Low | Low | High | High | High |
| 1993, Montejo | Low | Low | Low | High | High | High |
| 1994, Colmenero | High | Low | Low | High | High | High |
| 1995, Solera | High | Low | Low | High | High | High |
| 1996, Kalo | Some concerns | Low | Low | High | High | High |
| 1999, Agalar | Some concerns | Low | Low | High | High | High |
| 2002, Saltoglu | High | Low | High | High | High | High |
| 2004, Solera | Low | Low | High | High | High | High |
| 2004, Karabay | Some concerns | Low | Low | High | High | High |
| 2004, Roushan | Some concerns | Some concerns | Low | Some concerns | Some concerns | Some concerns |
| 2005, Ersoy | Some concerns | High | High | High | High | High |
| 2006, Roushan | Low | High | High | Some concerns | High | High |
| 2007, Alavi | Some concerns | High | High | Low | Some concerns | High |
| 2007, Ranjbar | Some concerns | High | High | High | High | High |
| 2009, Keramat | Some concerns | Low | Low | High | High | High |
| 2009, Sarmadian | Some concerns | High | High | High | High | High |
| 2010, Roushan | High | Low | Low | High | High | High |
| 2012, Hashemi | Some concerns | Low | High | High | High | High |
| 2014, Sofian | High | Low | Low | High | Some concerns | High |
| 2016, Hasanain | Some concerns | Low | Low | High | High | High |
| 2018, Majzoobi | Low | Low | Low | Low | Some concerns | Some concerns |
| 2020, Karami | Low | Low | Low | High | Low | High |
| 2022, Majzoobi | Low | Low | Low | High | Low | High |

**REFERENCE**

1. Ariza, J., Gudiol, F., Pallarés, R., Rufí, G. & Fernández-Viladrich, P. Comparative trial of co-trimoxazole versus tetracycline-streptomycin in treating human brucellosis. *Journal of infectious diseases* **152**, 1358–1359 (1985).
2. Rodriguez Zapata, M., Gamo Herranz, A. & De La Morena Fernández, J. Comparative study of two regimens in the treatment of brucellosis. *Chemioterapia* **6**, 360–362 (1987).
3. Acocella, G. *et al.* Comparison of three different regimens in the treatment of acute brucellosis: a multicenter multinational study. *J Antimicrob Chemother* **23**, 433–439 (1989).
4. Colmenero Castillo, J., Hernandez Marquez, S., Reguera Iglesias, J., Cabrera Franquelo, F., Rius Diaz, F., & Alonso, A *.* Comparative trial of doxycycline plus streptomycin versus doxycycline plus rifampin for the therapy of human brucellosis. *Chemotherapy* **35**, 146–152 (1989).
5. Solera, J., Medrano, F., Rodríguez, M., Geijo, P. & Paulino, J. [A comparative therapeutic and multicenter trial of rifampicin and doxycycline versus streptomycin and doxycycline in human brucellosis]. *Med Clin (Barc)* **96**, 649–653 (1991).
6. Ariza, J. *et al.* Treatment of human brucellosis with doxycycline plus rifampin or doxycycline plus streptomycin. A randomized, double-blind study. *Ann Intern Med* **117**, 25–30 (1992).
7. Akova, M., Uzun, O., Akalin, H. E., Hayran, M., Unal, S., & Gür, D*.* Quinolones in treatment of human brucellosis: comparative trial of ofloxacin-rifampin versus doxycycline-rifampin. *Antimicrob Agents Chemother* **37**, 1831–1834 (1993).
8. Montejo, J., Alberola, I., Glez-Zarate, P., Alvarez, A., Alonso, J., Canovas, A., & Aguirre, C. Open, randomized therapeutic trial of six antimicrobial regimens in the treatment of human brucellosis. *Clinical infectious diseases* **16**, 671–676 (1993).
9. Colmenero, J., Porras, J., Cárdenas, A., Ocón, P., Reguera, J., Delgado, M., & Sedeño, J*.* Evaluation of the Chromotitre EIA test for the diagnosis of human brucellosis. *Enferm Infecc Microbiol Clin* **12**, 60–65 (1994).
10. Solera, J. *et al.* Doxycycline-rifampin versus doxycycline-streptomycin in treatment of human brucellosis due to Brucella melitensis. *Antimicrob Agents Chemother* **39**, 2061–2067 (1995).
11. Kalo, T., Novi, S., Nushi, A. & Dedja, S. Ciprofloxacin plus doxycycline versus rifampicin plus doxycycline in the treatment of acute brucellosis. *Med Mal Infect* **26**, 587–589 (1996).
12. Agalar, C., Usubutun, S. & Turkyilmaz, R. Ciprofloxacin and rifampicin versus doxycycline and rifampicin in the treatment of brucellosis. *European journal of clinical microbiology & infectious diseases* **18**, 535–538 (1999).
13. Saltoglu, N., Tasova, Y., Inal, A. S., Seki, T. & Aksu, H. S. Efficacy of rifampicin plus doxycycline versus rifampicin plus quinolone in the treatment of brucellosis. *Saudi Med J* **23**, 921–924 (2002).
14. Solera, J. *et al.* A randomized, double-blind study to assess the optimal duration of doxycycline treatment for human brucellosis. *Clinical infectious diseases* **39**, 1776–1782 (2004).
15. Karabay, O., Sencan, I., Kayas, D. & Sahin, I. Ofloxacin plus rifampicin versus doxycycline plus rifampicin in the treatment of brucellosis: a randomized clinical trial [ISRCTN11871179]. *BMC Infect Dis* **4**, 18–18 (2004).
16. Roushan, M., Gangi, S. M. E. & Ahmadi, S. A. A. Comparison of the efficacy of two months of treatment with co-trimoxazole plus doxycycline vs. co-trimoxazole plus rifampin in brucellosis. *Swiss Med Wkly* **134**, 564–568 (2004).
17. Ersoy, Y., Sonmez, E., Tevfik, M. R. & But, A. D. Comparison of three different combination therapies in the treatment of human brucellosis. *Trop Doct* **35**, 210–212 (2005).
18. Roushan, M., Mohraz, M., Janmohammadi, N. & Hajiahmadi, M. Efficacy of cotrimoxazole and rifampin for 6 or 8 weeks of therapy in childhood brucellosis. *Pediatr Infect Dis J* **25**, 544–545 (2006).
19. Alavi, S. & Rajabzadeh, A. Comparison of two chemotherapy regimen: doxycycline-rifampicin and doxycycline cotrimoxazol in the brucellosis patients Ahvaz, Iran, 2004-2006. *Pak J Med Sci* **23**, 889–892 (2007).
20. Ranjbar, M. *et al.* Comparison between doxycycline-rifampin-amikacin and doxycycline-rifampin regimens in the treatment of brucellosis. *International Journal of Infectious Diseases* **11**, 152–156 (2007).
21. Keramat, F., Ranjbar, M., Mamani, M., Hashemi, S. H. & Zeraati, F. A comparative trial of three therapeutic regimens: ciprofloxacin-rifampin, ciprofloxacin-doxycycline and doxycycline-rifampin in the treatment of brucellosis. *Trop Doct* **39**, 207–210 (2009).
22. Sarmadian, H., Didgar, F., Sufian, M., Zarinfar, N. & Salehi, F. Comparison Between Efficacy of Cipofoxacin Doxycycline and Rifampin - Doxycycline Regimens in Treatment and Relapse of Brucellosis. *Tropical medicine & international health* **14**, 209–209 (2009).
23. Roushan, M., Amiri, M., Janmohammadi, N., Hadad, M., Javanian, M., Baiani, M., & Bijani, A. Comparison of the efficacy of gentamicin for 5 days plus doxycycline for 8 weeks versus streptomycin for 2 weeks plus doxycycline for 45 days in the treatment of human brucellosis: a randomized clinical trial. *J Antimicrob Chemother* **65**, 1028–1035 (2010).
24. Hashemi, S. *et al.* Comparison of doxycycline-streptomycin, doxycycline-rifampin, and ofloxacin-rifampin in the treatment of brucellosis: a randomized clinical trial. *International journal of infectious diseases* **16**, e247-51 (2012).
25. Sofian, M. *et al.* Comparison of two durations of triple-drug therapy in patients with uncomplicated brucellosis: A randomized controlled trial. *Scand J Infect Dis* **46**, 573–577 (2014).
26. Hasanain, A., Mahdy, R., Mohamed, A. & Ali, M. A randomized, comparative study of dual therapy (doxycycline-rifampin) versus triple therapy (doxycycline-rifampin-levofloxacin) for treating acute/subacute brucellosis. *Brazilian Journal of Infectious Diseases* **20**, 250–254 (2016).
27. Majzoobi, M. *et al.* Effect of hydroxychloroquine on treatment and recurrence of acute brucellosis: a single-blind, randomized clinical trial. *Int J Antimicrob Agents* **51**, 365–369 (2018).
28. Karami, A., Mobaien, A., Jozpanahi, M., Moghtader-Mojdehi, A. & Javaheri, M. Effect of 8-week and 12-week triple therapy (doxycycline, rifampicin, and gentamicin) on brucellosis: A comparative study. *Journal of Acute Disease* **9**, 161–165 (2020).
29. Majzoobi, M. M., Hashmi, S. H., Emami, K. & Soltanian, A. R. Combination of doxycycline, streptomycin and hydroxychloroquine for short-course treatment of brucellosis: a single-blind randomized clinical trial. *Infection* **50**, 1267–1271 (2022).
