# Supplementary material for: Efficacy and safety of therapeutic strategies for human brucellosis: A systematic review and network meta-analysis
Source: PLoS Negl Trop Dis. 2024 Mar 11;18(3):e0012010. doi: 10.1371/journal.pntd.0012010 (PMC10978012; doi:10.1371/journal.pntd.0012010)
Supplement: S7 Table — (DOCX) [file pntd.0012010.s007.docx]

**S7_Table**_Risk of bias for adverse events outcome.

| **Year, Author** | **Domain 1** | **Domain 2** | **Domain 3** | **Domain 4** | **Domain 5** | **Risk of bias** |
| --- | --- | --- | --- | --- | --- | --- |
| 1987, Rodriguez Zapata | High | Low | Low | High | High | High |
| 1989, Acocella | Low | Low | Low | High | High | High |
| 1989, Colmenero | High | Low | Low | High | High | High |
| 1995, Solera | High | Low | Low | High | High | High |
| 1999, Agalar | Some concerns | Low | Low | High | High | High |
| 2004, Solera | Low | Low | High | High | High | High |
| 2005, Ersoy | Some concerns | Some concerns | High | High | High | High |
| 2007, Ranjbar | Some concerns | High | High | Low | Some concerns | High |
| 2009, Keramat | Some concerns | Low | Low | High | High | High |
| 2010, Roushan | High | Low | Low | High | High | High |
| 2012, Hashemi | Some concerns | Low | High | High | High | High |
| 2016, Hasanain | Some concerns | Low | Low | High | High | High |
| 2018, Majzoobi | Low | Low | Low | High | High | High |

**REFERENCE**

1. Rodriguez Zapata, M., Gamo Herranz, A. & De La Morena Fernández, J. Comparative study of two regimens in the treatment of brucellosis. *Chemioterapia* **6**, 360–362 (1987).
2. Acocella, G. *et al.* Comparison of three different regimens in the treatment of acute brucellosis: a multicenter multinational study. *J Antimicrob Chemother* **23**, 433–439 (1989).
3. Colmenero Castillo, J., Hernandez Marquez, S., Reguera Iglesias, J., Cabrera Franquelo, F., Rius Diaz, F., & Alonso, A *.* Comparative trial of doxycycline plus streptomycin versus doxycycline plus rifampin for the therapy of human brucellosis. *Chemotherapy* **35**, 146–152 (1989).
4. Solera, J. *et al.* Doxycycline-rifampin versus doxycycline-streptomycin in treatment of human brucellosis due to Brucella melitensis. *Antimicrob Agents Chemother* **39**, 2061–2067 (1995).
5. Agalar, C., Usubutun, S. & Turkyilmaz, R. Ciprofloxacin and rifampicin versus doxycycline and rifampicin in the treatment of brucellosis. *European journal of clinical microbiology & infectious diseases* **18**, 535–538 (1999).
6. Solera, J. *et al.* A randomized, double-blind study to assess the optimal duration of doxycycline treatment for human brucellosis. *Clinical infectious diseases* **39**, 1776–1782 (2004).
7. Ersoy, Y., Sonmez, E., Tevfik, M. R. & But, A. D. Comparison of three different combination therapies in the treatment of human brucellosis. *Trop Doct* **35**, 210–212 (2005).
8. Ranjbar, M. *et al.* Comparison between doxycycline-rifampin-amikacin and doxycycline-rifampin regimens in the treatment of brucellosis. *International Journal of Infectious Diseases* **11**, 152–156 (2007).
9. Keramat, F., Ranjbar, M., Mamani, M., Hashemi, S. H. & Zeraati, F. A comparative trial of three therapeutic regimens: ciprofloxacin-rifampin, ciprofloxacin-doxycycline and doxycycline-rifampin in the treatment of brucellosis. *Trop Doct* **39**, 207–210 (2009).
10. Roushan, M., Amiri, M., Janmohammadi, N., Hadad, M., Javanian, M., Baiani, M., & Bijani, A. Comparison of the efficacy of gentamicin for 5 days plus doxycycline for 8 weeks versus streptomycin for 2 weeks plus doxycycline for 45 days in the treatment of human brucellosis: a randomized clinical trial. *J Antimicrob Chemother* **65**, 1028–1035 (2010).
11. Hashemi, S. *et al.* Comparison of doxycycline-streptomycin, doxycycline-rifampin, and ofloxacin-rifampin in the treatment of brucellosis: a randomized clinical trial. *International journal of infectious diseases* **16**, e247-51 (2012).
12. Hasanain, A., Mahdy, R., Mohamed, A. & Ali, M. A randomized, comparative study of dual therapy (doxycycline-rifampin) versus triple therapy (doxycycline-rifampin-levofloxacin) for treating acute/subacute brucellosis. *Brazilian Journal of Infectious Diseases* **20**, 250–254 (2016).
13. Majzoobi, M. *et al.* Effect of hydroxychloroquine on treatment and recurrence of acute brucellosis: a single-blind, randomized clinical trial. *Int J Antimicrob Agents* **51**, 365–369 (2018).
