# Supplementary material for: Efficacy and safety of therapeutic strategies for human brucellosis: A systematic review and network meta-analysis
Source: PLoS Negl Trop Dis. 2024 Mar 11;18(3):e0012010. doi: 10.1371/journal.pntd.0012010 (PMC10978012; doi:10.1371/journal.pntd.0012010)
Supplement: S8 Table — (DOCX) [file pntd.0012010.s008.docx]

**S8_Table**_Risk of bias for time to defervescence outcome.

| **Year, Author** | **Domain 1** | **Domain 2** | **Domain 3** | **Domain 4** | **Domain 5** | **Risk of bias** |
| --- | --- | --- | --- | --- | --- | --- |
| 1985, Ariza | High | Low | Low | High | Some concerns | High |
| 1987, Rodriguez Zapata | High | Low | Low | High | Some concerns | High |
| 1995, Solera | High | Low | Low | Low | Some concerns | High |
| 1999, Agalar | Some concerns | Low | Low | High | Some concerns | High |
| 2004, Karabay | Some concerns | Low | Low | Low | Some concerns | Some concerns |

**REFERENCE**

1. Ariza, J., Gudiol, F., Pallarés, R., Rufí, G. & Fernández-Viladrich, P. Comparative trial of co-trimoxazole versus tetracycline-streptomycin in treating human brucellosis. *Journal of infectious diseases* **152**, 1358–1359 (1985).
2. Rodriguez Zapata, M., Gamo Herranz, A. & De La Morena Fernández, J. Comparative study of two regimens in the treatment of brucellosis. *Chemioterapia* **6**, 360–362 (1987).
3. Solera, J. *et al.* Doxycycline-rifampin versus doxycycline-streptomycin in treatment of human brucellosis due to Brucella melitensis. *Antimicrob Agents Chemother* **39**, 2061–2067 (1995).
4. Agalar, C., Usubutun, S. & Turkyilmaz, R. Ciprofloxacin and rifampicin versus doxycycline and rifampicin in the treatment of brucellosis. *European journal of clinical microbiology & infectious diseases* **18**, 535–538 (1999).
5. Karabay, O., Sencan, I., Kayas, D. & Sahin, I. Ofloxacin plus rifampicin versus doxycycline plus rifampicin in the treatment of brucellosis: a randomized clinical trial [ISRCTN11871179]. *BMC Infect Dis* **4**, 18–18 (2004).
